# Association of tissue lymphocyte immunophenotype and clinical outcomes: A prospective study in patients with ulcerative colitis treated with vedolizumab

**DOI:** 10.1371/journal.pone.0340271

**Published:** 2026-02-03

**Authors:** Katsuyoshi Ando, Shin Kashima, Aki Sakatani, Hiroaki Konishi, Atsuo Maemoto, Takahiro Ito, Masaki Taruishi, Kenichi Hamagami, Kaori Ishiguro, Mikihiro Fujiya

**Affiliations:** 1 Division of Gastroenterology, Department of Internal Medicine, Asahikawa Medical University, Asahikawa, Hokkaido, Japan; 2 Department of Gastroenterology and Advanced Medical Sciences, Asahikawa Medical University, Asahikawa, Hokkaido, Japan; 3 Inflammatory Bowel Disease Center, Sapporo Higashi Tokushukai Hospital, Sapporo, Hokkaido, Japan; 4 Department of Gastroenterology, Asahikawa City Hospital, Asahikawa, Hokkaido, Japan; 5 Japan Medical Office, Takeda Pharmaceutical Company Limited, Chuo-ku, Tokyo, Japan; University of Missouri, UNITED STATES OF AMERICA

## Abstract

**Introduction:**

Vedolizumab binds to α4β7 integrin, thereby inhibiting lymphocyte migration into the gastrointestinal tract. It is used to treat moderate to severe ulcerative colitis (UC) and Crohn’s disease. This study evaluated vedolizumab concentrations, α4β7 integrin saturation, and T lymphocyte immunophenotype proportions in blood, serum and inflamed colorectal tissue according to treatment efficacy in patients with moderate to severe UC.

**Methods:**

This was a phase 4, multicenter, open-label, single-arm study. Patients were observed for a total of 54 weeks. Clinical remission was defined as complete Mayo score ≤ 2 or partial Mayo score ≤ 1.

**Results:**

The study included 11 patients with UC, 10 of whom were tumor necrosis factor alpha antagonist therapy-naïve. Seven and six patients were in remission at weeks 14 and 54, respectively. Vedolizumab concentrations and lymphocyte α4β7 integrin saturation in serum and inflamed colorectal tissue at weeks 14 and 54 did not differ significantly between remitters and non-remitters. The proportion of T cell subsets differed in remitters and non-remitters for CD4^+^ T cells in blood at week 14 (62.7% vs 47.4%, *p* = 0.0061) and CD161^+^ memory CD4^+^ T cells (65.7% vs 53.1%, *p* = 0.0357) in inflamed colorectal tissue at week 54. Week 54 remitters had higher proportions of CD161^+^ memory CD4^+^ T cells in inflamed colorectal tissue at baseline (before vedolizumab treatment) than did week 54 non-remitters (48.5% vs 31.3%, *p* = 0.0303).

**Conclusion:**

T cell immunophenotype may be a promising predictive biomarker of vedolizumab treatment efficacy.

## Introduction

Ulcerative colitis (UC) is a chronic, immune-mediated inflammatory bowel disease characterized by recurrent flare-ups and remissions. Recent studies suggest that the underlying disease pathophysiology involves a breakdown of immune tolerance to intraluminal antigens (including intestinal bacteria), colorectal tissue infiltration by inflammatory T lymphocytes, and aberrant production of pro- and anti-inflammatory cytokines [1,2].

Vedolizumab is a recombinant humanized immunoglobulin G1 monoclonal antibody that specifically binds to the lymphocyte plasma membrane protein α4β7 integrin, thereby blocking the interaction between α4β7 integrin and its primary ligand, mucosal addressin cell adhesion molecule-1 (MAdCAM-1) [3–5]. By binding to α4β7 integrin, vedolizumab inhibits the migration of lymphocytes to the intestinal mucosa and gut-associated lymphoid tissue [6].

The efficacy and safety profiles of vedolizumab were demonstrated in phase 3 clinical studies in patients with moderate to severe UC [7,8]. In the GEMINI 1 study, higher vedolizumab trough serum concentrations were associated with higher rates of clinical remission at week 6 (end of induction) and week 52 (maintenance) [7,9]. Some individuals with UC have marked intestinal inflammation despite adequate vedolizumab serum concentrations and full saturation of α4β7 integrin on peripheral lymphocytes [10]. It may be that the vedolizumab dose–response relationship is more accurately reflected by vedolizumab concentrations and α4β7 integrin saturation in intestinal tissue rather than in serum [11]. Identifying predictive tissular biomarkers of response to vedolizumab therapy would be useful to guide treatment decisions in clinical practice.

The current study evaluated vedolizumab concentrations, α4β7 integrin saturation, and T cell immunophenotype proportions in blood, serum, and colorectal tissue in relation to treatment efficacy in patients with moderate to severe UC. A study hypothesis was that phenotype at baseline and changes to phenotype during treatment may be related to the therapeutic effect of vedolizumab. The study aimed to increase understanding of the contribution of different α4β7-expressing cells in blood and tissue at baseline and during treatment to vedolizumab treatment efficacy and their potential utility as predictive biomarkers for treatment efficacy with vedolizumab in real-world clinical practice.

## Methods

### Study design

This was a phase 4, multicenter, open-label, single-arm study to evaluate the relationship between vedolizumab concentrations in serum and colorectal tissue, α4β7 receptor saturation, and clinical outcomes in patients with moderately to severely active UC treated with vedolizumab (Vedolizumab-4026; Japan Registry of Clinical Trials ID: jRCTs011200009). Patients received intravenous vedolizumab 300 mg at weeks 0, 2, and 6 as induction therapy and every 8 weeks thereafter as maintenance therapy for a total treatment period of 54 weeks. Outcome assessments were performed at weeks 14 and 54.

The study was conducted in accordance with the Declaration of Helsinki and the International Council for Harmonisation and Good Clinical Practice guidelines for the conduct of clinical research. All participants provided written informed consent prior to initiation of any study procedures. The first and last informed consent were obtained on 8 April 2021 and 11 March 2022, respectively. Capacity to consent was assessed by site investigators based on the patient’s ability to understand the study’s nature, objectives, risks, and benefits, as outlined in the approved informed consent form. The study and the consent process were approved by the Hokkaido University Certified Review Board (approval no. 020−004). The full study protocol is provided as supporting information (S1 File).

### Study population

Eligible patients were aged 20–80 years at informed consent, were diagnosed with UC at least 3 months prior to enrollment, had moderately to severely active disease as defined by a complete Mayo score of 6–12 and an endoscopic subscore of 2 or higher within 10 days before the first dose of vedolizumab, and had experienced prior treatment failure with corticosteroids, immunomodulators (azathioprine or 6-mercaptopurine), or tumor necrosis factor alpha (TNFα) antagonists.

Patients were ineligible if they had received ustekinumab or infliximab within 8 weeks, golimumab within 4 weeks, adalimumab within 2 weeks, or a Janus kinase inhibitor within 1 week before the planned first dose of vedolizumab, or if they had been treated with vedolizumab, natalizumab, efalizumab, or rituximab. Other main exclusion criteria were UC limited to the rectum only, history of extensive colonic resection, subtotal or total colectomy, history of ileostomy, colostomy, or known symptomatic stenosis of the intestine, active infection within 1 month before the first administration of vedolizumab, current or past history of malignant tumor, history of hypersensitivity or allergies to vedolizumab or any vedolizumab excipient, and other criteria that in the opinion of the investigator would prohibit study participation.

### Outcome assessments

The primary outcome was the relationship between vedolizumab concentrations in serum and colorectal tissue and clinical remission (defined as a complete Mayo score ≤ 2 or partial Mayo score ≤ 1) at week 54. Secondary outcomes included α4β7 receptor saturation and T cell immunophenotype at weeks 14 and 54, and T cell immunophenotype at baseline (before vedolizumab treatment initiation) in blood and in inflamed colorectal tissue. Secondary outcomes were assessed separately in patients with and without clinical remission. Exploratory outcomes included concentrations of fecal calprotectin (measured by EliA Calprotectin 2 immunoassay [Phadia 250; Thermo Fisher Scientific, Waltham, MA, USA]) and soluble MAdCAM-1 (sMAdCAM-1; measured by enzyme-linked immunosorbent assay [Hycult Biotech Inc., Wayne, PA, USA]) at baseline, week 14, and week 54.

### Blood, serum, and tissue sample collection

Serum and inflamed colorectal tissue samples were collected at weeks 14 and 54 for determining vedolizumab concentrations. Blood and inflamed colorectal tissue samples were collected at baseline week 14 and week 54 for fluorescence-activated cell sorting (FACS) analysis. Colorectal endoscopy with biopsy sample collection could be undertaken within 9 days before each time point. Serum and tissue samples were processed and frozen immediately after collection.

### Vedolizumab concentrations in serum and tissue

Blood samples were incubated for approximately 30 minutes at room temperature and centrifuged at 1500 × g for 10 minutes at 4°C. Colorectal tissue was homogenized in lysis buffer (50 mM Tris, pH 7.4, containing 100 mM NaCl, 0.1% Triton X-100, 1 mM EDTA, and Halt protease inhibitor) and centrifuged at 12 000 × g for 10 minutes at 4°C. Supernatants were diluted with lysis buffer to a total protein concentration of 3.0 mg/mL, measured using Pierce BCA protein assay (Thermo Fisher Scientific, Waltham, MA, USA). Vedolizumab concentrations were measured by enzyme-linked immunosorbent assay at QPS Holdings, LLC (Newark, DE, USA). Anti-idiotype MLN0002 antibody was used as coating and horseradish peroxidase-conjugated mouse anti-human immunoglobulin G was used for detection, allowing the detection of vedolizumab concentrations from 0.2 µg/mL to 8.0 µg/mL in serum samples and from 25 ng/mL to 2500 ng/mL in tissue samples.

### Flow cytometric analysis

Blood-derived cells were sedimented from blood samples. Lamina propria mononuclear cells were prepared from colorectal tissue: colorectal tissue was agitated with digestion mix containing collagenase (100 units/mL, Sigma-Aldrich, St. Louis, MO, USA) and DNase (4.5–7 units/mL, Nippon Gene, Toyama, Japan), incubated for 1 hour at 37°C, pressed through a 100 µm cell strainer (Greiner Bio-One, Kremsmünster, Austria), washed in phosphate buffered saline by centrifugation at 600 × g for 10 minutes at room temperature. Cells were stained with labeled antibodies against CD3, CD4, CD8, CD25, CD45, CD45RO, CD127, CD161, and γδ T cell receptor (TCR) (S1 Table), collected by centrifugation at 300 × g for 5 minutes at 4°C, and incubated for 30 minutes at 2–8°C with phycoerythrin-conjugated streptavidin diluted in FACS buffer containing 1% fetal bovine serum (0.03 µg cells/50 μL/tube). Cells were subsequently washed twice by addition of FACS buffer and centrifugation at 300 × g for 5 minutes at 4°C, followed by resuspension in FACS buffer. Flow cytometry was conducted using a BD LSRFortessa™ Cell Analyzer (BD Biosciences, Franklin Lakes, NJ, USA), and data were analyzed using FlowJo™ software v10. Gating strategy is shown in S1 Fig.

T cells were classified according to immunophenotype, and T cell phenotype proportions (%) were determined as a proportion of each parent population (S2 Table). Lymphocyte α4β7 integrin saturation (%) was estimated with the anti-α4β7 antibody ACT-1 separately for each immunophenotype as follows: (1 – [proportion of parent population at week 14 or week 54/ proportion of parent population at baseline]) × 100.

### Statistical analyses

Data were represented as counts (proportions) for categorical variables. Median (interquartile range [IQR]) were used for continuous variables. Mann-Whitney U-test was performed to compare variables between patients with and without clinical remission. Individuals with missing data on Mayo scores, vedolizumab concentrations in colorectal tissue or serum, α4β7 integrin saturation, biomarkers, or immunophenotypes of various lymphocytes were excluded from the analyses at that time point; individuals with missing data were those who discontinued treatment or who did not undergo an endoscopy.

## Results

### Study population

**Fig 1:**
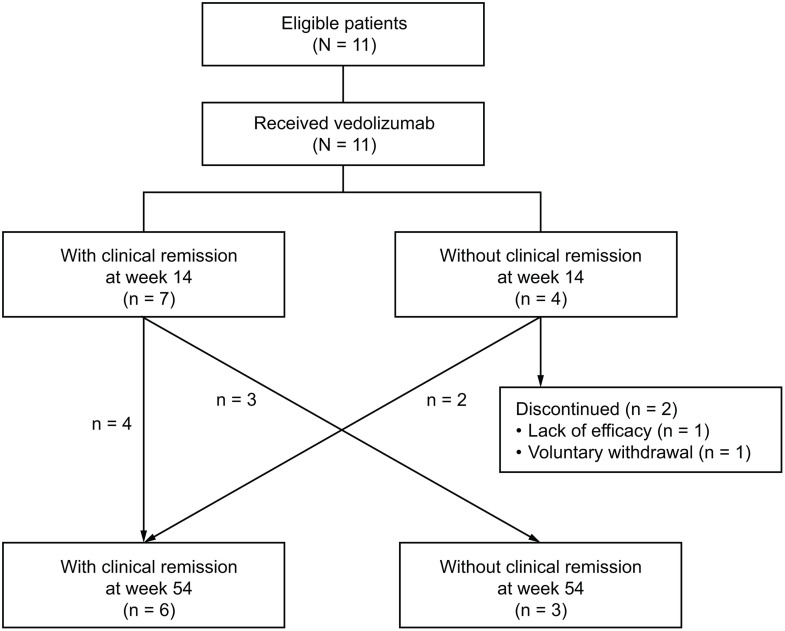
Patient disposition. All eligible patients received vedolizumab as induction and maintenance therapy. Outcome assessments were performed at weeks 14 and 54. In total, 11 patients were included for analysis at week 14 (n = 7 with clinical remission, n = 4 without clinical remission) and 9 patients at week 54 (n = 6 with clinical remission, n = 3 without clinical remission).

The study was conducted between 8 April 2021 and 6 February 2023 and included a total of 11 patients with UC (Fig 1). Baseline demographic and clinical characteristics are summarized in Table 1. The median age was 54 years (IQR, 40–67), median (IQR) complete Mayo score was 7.0 (7.0–8.0), and median (IQR) partial Mayo score (i.e., excluding the endoscopy subscore) was 5.0 (5.0–5.0). Ten patients were TNFα antagonist-naïve and one patient had previously received TNFα antagonist therapy. Ten patients had a history of corticosteroid use and four patients had a history of immunomodulator use. Seven patients had a disease duration of 7 or more years.

**Table 1:** Baseline demographic and clinical characteristics.

Patient characteristics	All patients (N = 11)
Age, years,^a^ median (IQR)	54 (40–67)
Male sex, n (%)	6 (55)
BMI, kg/m^2^, median (IQR)	24.9 (20.9–28.1)
Disease duration, years, n (%)	
< 1	3 (27)
1 to < 3	1 (9)
3 to < 7	0 (0)
≥ 7	7 (64)
Complete Mayo score, median (IQR)	7.0 (7.0–8.0)
Partial Mayo score, median (IQR)	5.0 (5.0–5.0)
Extent of disease, n (%)	
Total colitis	8 (73)
Left-sided colitis	3 (27)
Extraintestinal complications, n (%)	1 (9)
Biologic-naïve, n (%)	10 (91)
Past UC treatment and reason for cessation, n (%)	
Corticosteroids	10 (91)
Resistance	5 (45)
Dependence	4 (36)
Intolerance	1 (9)
Immunomodulators^b^	4 (36)
Refractory	2 (18)
Intolerance	2 (18)
TNFα antagonists	1 (9)
Inadequate response	1 (9)

^a^ At the time of informed consent.

^b^ Azathioprine, 6-mercaptopurine.

BMI, body mass index; IQR, interquartile range; TNFα, tumor necrosis factor alpha; UC, ulcerative colitis.

### Clinical outcomes

Seven patients were in clinical remission at week 14 (Fig 1). Two of the four patients without clinical remission discontinued the study between week 14 and week 54, and the two other patients transitioned to the remission group by week 54 (slow responders). Three patients who achieved clinical remission at week 14 subsequently transitioned to the non-remission group by week 54 (loss of response). In total, six patients were in clinical remission at week 54 and three patients were not.

**Fig 2:**
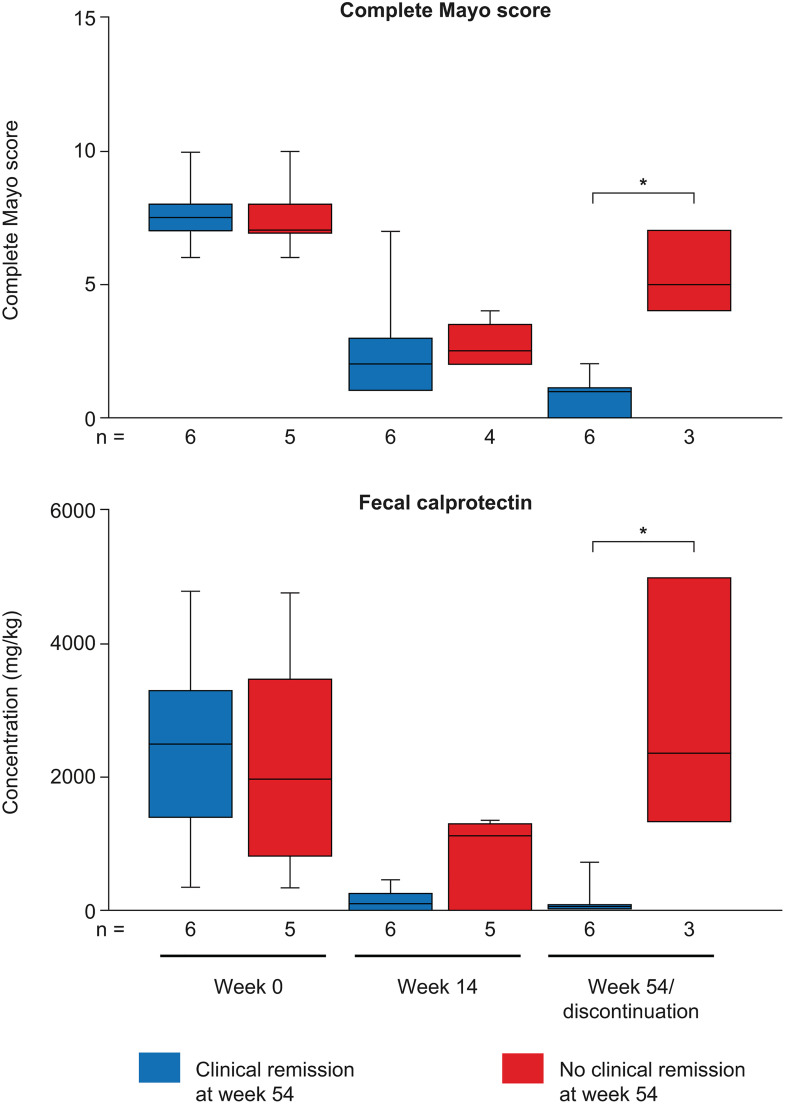
Complete Mayo score and fecal calprotectin levels. Box plots showing the complete Mayo score and fecal calprotectin levels at baseline, week 14, and week 54 in patients with and without clinical remission at week 54. In the box plots, horizontal lines depict the Q1–Q3 range and median, and whiskers represent the minimum and maximum values. Graphs are based on observed data; patients with missing data were excluded from analyses. *p < 0.05. Q, quartile.

The complete Mayo scores in patients with week 54 remission and week 54 non-remission were similar at baseline (median [IQR]: 7.5 [7.0–8.0] vs 7.0 [7.0–8.0]) and at week 14 (median [IQR]: 2.0 [1.0–3.0] vs 2.5 [2.0–3.5]) (Fig 2, S3 Table, S2 File). At week 54, the median (IQR) complete Mayo score was 1.0 (0–1.0) in patients with remission and 5.0 (4.0–7.0) in those without remission (*p* = 0.0119). Fecal calprotectin levels in patients with and without week 54 remission were similar at baseline (median [IQR] 2510 [1410–3320] mg/kg vs 1990 [821–3500] mg/kg, respectively; Fig 2, S4 Table, S2 File). At week 54, fecal calprotectin levels were significantly different between patients with and without week 54 remission (median [IQR]: 52 [10–114] vs 2380 [1340–5010] mg/kg; *p* = 0.0238). Serum sMAdCAM-1 concentrations declined quickly after treatment and were similar in patients with and without week 54 remission (S2 Fig, S4 Table, S2 File).

### Vedolizumab concentrations and cytometry according to clinical remission

Serum vedolizumab concentrations and cytometry were available for seven and four patients in week 14 remission and non-remission, and for six and three patients in week 54 remission and non-remission, respectively. Tissue values for vedolizumab concentrations and cytometry were available for seven and three patients in week 14 remission and non-remission, and for five (except for vedolizumab concentration, n = 4) and three patients in week 54 remission and non-remission, respectively.

#### Vedolizumab concentrations.

**Fig 3:**
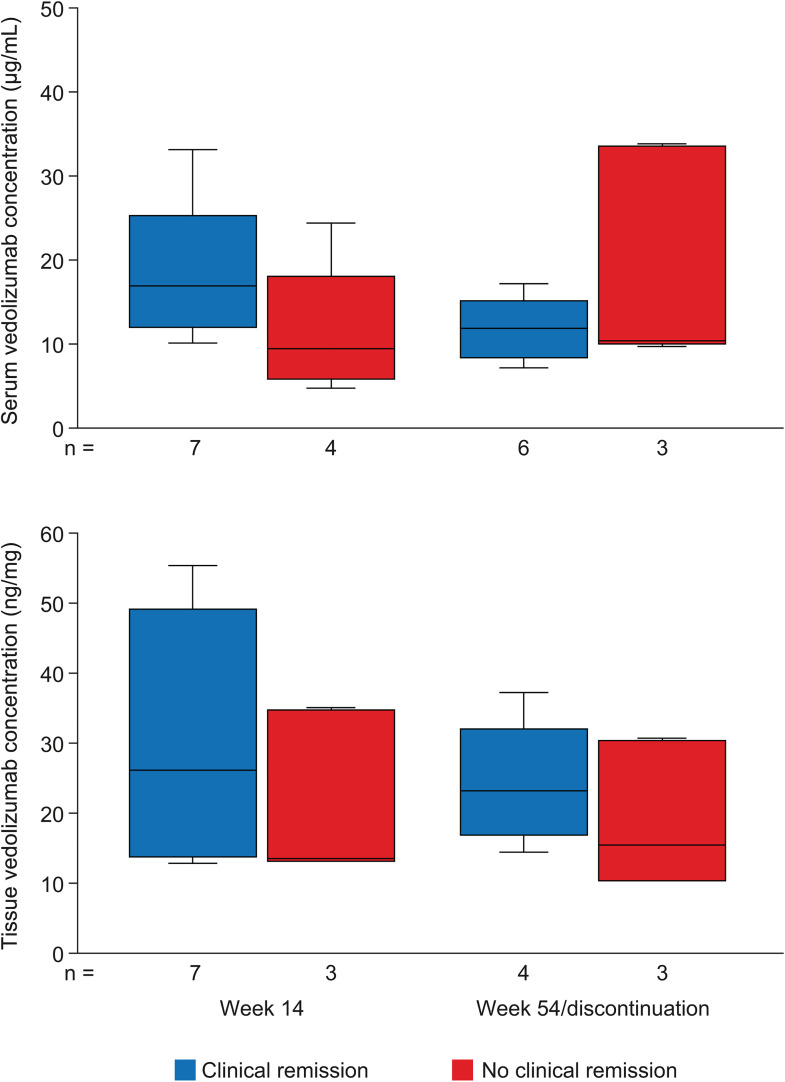
Vedolizumab concentrations in serum and colorectal tissue. Box plots showing the concentrations of vedolizumab in serum and colorectal tissue at week 14 and week 54/discontinuation in patients with and without clinical remission. In the box plots, horizontal lines depict the Q1–Q3 range and median, and whiskers represent the minimum and maximum values. Q, quartile.

Fig 3 depicts vedolizumab concentrations in serum and inflamed colorectal tissue at week 14 according to remission at week 14 and at week 54 according to remission at week 54. Vedolizumab concentrations were numerically higher in patients with than without remission at week 14 in serum (median [IQR]: 17.0 [12.1–25.3] vs 9.5 [6.0–18.1] µg/mL) and in tissue (median [IQR]: 26.1 [13.7–49.0] vs 13.4 [13.2–34.7] ng/mg), and at week 54 in tissue (median [IQR]: 23.2 [16.9–32.2] vs 15.4 [10.4–30.5] ng/mg) but not in serum (median [IQR]: 11.9 [8.6–15.3] vs 10.4 [10.1–33.7] µg/mL); however, differences between patients with and without remission were not statistically significant (S5 Table, S2 File).

#### Lymphocyte α4β7 integrin saturation.

**Fig 4:**
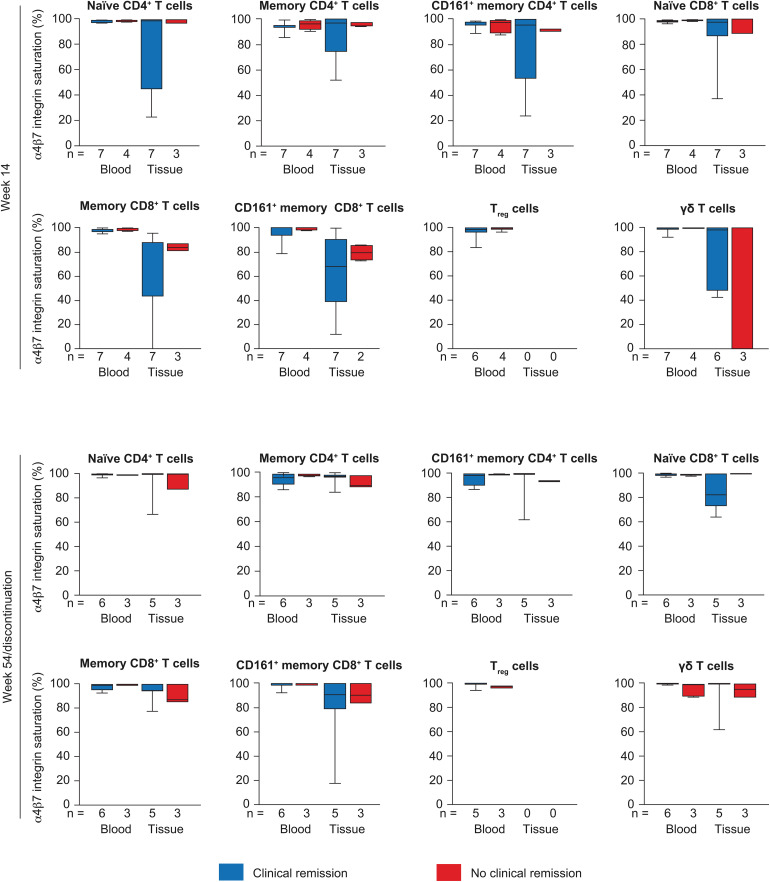
α4β7 integrin saturation. Median (IQR) α4β7 integrin saturation on lymphocytes in blood and colorectal tissue (%) at week 14 and week 54/discontinuation in patients with and without clinical remission. In the box plots, horizontal lines depict the Q1–Q3 range and median, and whiskers represent the minimum and maximum values. IQR, interquartile range; Q, quartile.

Fig 4 shows lymphocyte α4β7 integrin saturation in blood and inflamed colorectal tissue at week 14 according to remission at week 14 and at week 54 according to remission at week 54. Blood lymphocyte α4β7 integrin saturation was close to 100% at week 14 and at week 54. At week 54, tissue lymphocyte α4β7 integrin saturation in memory CD4^+^ T cells and memory CD8^+^ T cells was numerically higher in patients with remission versus those without remission; however, differences were not statistically significant (S6 Table, S2 File).

#### T cell immunophenotype proportions.

**Fig 5:**
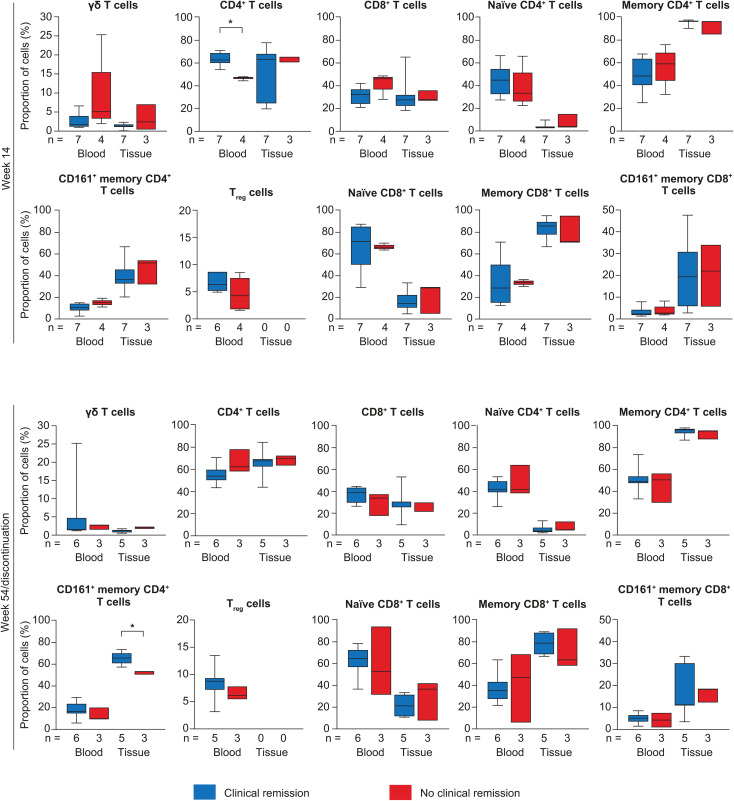
T cell immunophenotype proportions after vedolizumab treatment. Comparisons of median T cell immunophenotype proportions (%) after vedolizumab treatment at week 14 and week 54 between patients with and without clinical remission. Proportions were compared in patients with and without clinical remission using the Mann-Whitney U-test. T_reg_ cells could not be measured in tissue because of low quantities. In the box plots, horizontal lines depict the Q1–Q3 range and median, and whiskers represent the minimum and maximum values. *p < 0.05. Q, quartile.

Baseline disease severity was similar in patients with and without week 54 clinical remission and was thus not related to baseline differences in T cell immunophenotype proportions. Fig 5 depicts T cell immunophenotype proportions in blood and inflamed colorectal tissue at week 14 according to remission at week 14 and at week 54 according to remission at week 54. The proportion of T cell subsets differed significantly in patients with and without remission for CD4^+^ T cells in blood at week 14 (62.7% vs 47.4%, *p* = 0.0061) and CD161^+^ memory CD4^+^ T cells in inflamed colorectal tissue at week 54 (65.7% vs 53.1%, *p* = 0.0357) (S7 Table, S2 File). Similarly, in patients who experienced a loss of clinical remission from week 14 to week 54, CD161^+^ memory CD4^+^ T cells in inflamed colorectal tissue at week 54 were lower than in patients who had clinical remission at week 54 (S8 Table, S2 File).

**Fig 6:**
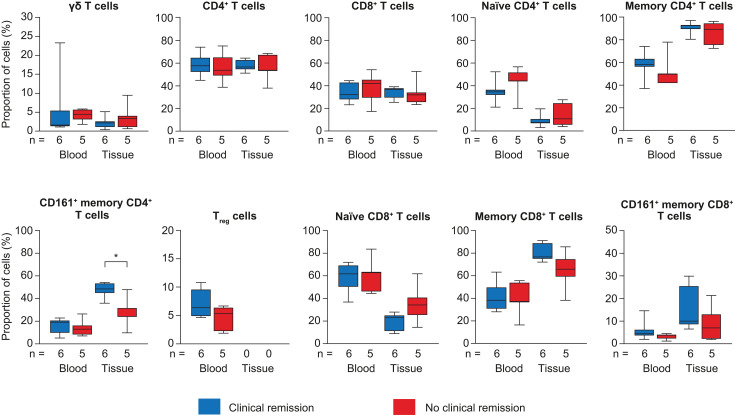
T cell immunophenotype proportions at baseline. Comparisons of T cell immunophenotype proportions (%) at baseline in patients with and without week 54 clinical remission. Proportions were compared in patients with and without clinical remission using the Mann-Whitney U-test. T_reg_ cells could not be measured in tissue because of low quantities. In the box plots, horizontal lines depict the Q1–Q3 range and median, and whiskers represent the minimum and maximum values. *p < 0.05. Q, quartile.

T cell immunophenotype proportions in blood and inflamed colorectal tissue at baseline (before vedolizumab treatment) according to remission at week 54 are depicted in Fig 6 and S7 Table. T cell immunophenotype proportions at baseline were significantly higher in patients with than without remission for CD161^+^ memory CD4^+^ T cells in inflamed colorectal tissue (48.5% vs 31.3%, *p* = 0.0303).

## Discussion

This prospective study assessed vedolizumab concentrations, α4β7 integrin saturation, and the role of different α4β7-expressing cells in blood and inflamed colorectal tissue in patients with UC experiencing (vs not experiencing) remission with vedolizumab. No statistically significant differences were observed between patients with and without remission in terms of vedolizumab serum and tissue concentrations or α4β7 integrin saturation at weeks 14 and 54. The small sample size may have reduced the power of the study to detect statistical significance. However, patients with remission had numerically higher vedolizumab serum and tissue concentrations at week 14 and numerically higher vedolizumab tissue (but not serum) concentrations at week 54 than those not in remission.

Several observational studies have identified potential thresholds for efficacy to guide therapeutic drug monitoring [12–16]. An association between vedolizumab serum trough concentrations and efficacy was reported in several real-world studies [12,14,16]. However, other data indicated that insufficient tissue exposure does not explain non-efficacy in patients with adequate serum vedolizumab concentrations [17]. We investigated whether vedolizumab tissue concentrations may be low even when serum concentrations are adequate and, furthermore, whether tissue concentrations might be more indicative than serum concentrations of clinical therapeutic effect of vedolizumab. Although we did not include early assessment of vedolizumab serum trough concentrations at weeks 2 or 6, our week 14 assessments showed numerically higher vedolizumab serum concentrations in patients with than without remission (17.0 vs 9.5 µg/mL; difference not statistically significant). These results are consistent with a previously reported potential threshold for therapeutic effect [12,14,18].

Efficient and sustained inhibition of lymphocyte trafficking to gut mucosal tissues requires immediate binding to and inhibition of newly expressed α4β7 integrin on activated T cells [3]. In the GEMINI 1 study, vedolizumab treatment resulted in more than 95% saturation of α4β7 integrin on CD4^+^CD45RO^+^ T cells in the peripheral circulation [7]. However, even when α4β7 integrin saturation is sufficient and vedolizumab serum trough concentrations are high, approximately 30% of patients with inflammatory bowel disease do not respond to vedolizumab treatment [12]. Thus, α4β7 integrin saturation, when measured alone, is insufficient to predict clinical outcome. We measured T cell immunophenotype proportions in tissue and blood to assess if the effect of vedolizumab on the mucosal immune system is qualitative rather than quantitative (i.e., affecting the proportions rather than the total numbers of cells) [19]. Patients with remission had significantly higher proportions of CD4^+^ T cells in blood at week 14 (62.7% vs 47.4%) and higher proportions of CD161^+^ memory CD4^+^ T cells in inflamed colorectal tissue at week 54 (65.7% vs 53.1%) than did those without remission. CD161 is implicated in autoimmune diseases such as psoriasis and Crohn’s disease [20–22], and is highly expressed on CD4^+^ T helper 17 (Th17) cells, which produce interleukin-17 (IL-17) [20,23–25]. A CD4^+^ tissue-resident memory T cell subset that expresses CD103, CCR5, and CD161 resides in the inflammatory intestinal mucosa of patients with Crohn’s disease [22], and CD161 may facilitate the transendothelial migration of CD161^+^ T cells [26]. Notably, in the current study, the proportions of CD161^+^ memory CD4^+^ T cells in inflamed colorectal tissue at baseline differed significantly between patients with and without remission (48.5% vs 31.3%), suggesting that pre-treatment CD161 expression on T cell subsets could be a biomarker of treatment efficacy based on vedolizumab-specific mechanisms of action.

We also compared the T cell immunophenotype in patients who initially achieved remission at week 14 but subsequently experienced loss of response by week 54 with those who maintained remission. Although CD161 ⁺ memory CD4 ⁺ T cell proportions in inflamed colorectal tissue were significantly lower in patients with a loss of response at week 54, no differences were observed at baseline or week 14, suggesting that this marker may reflect inflammation rather than predict loss of response. Interestingly, vedolizumab tissue concentrations were higher in those with a loss of response at week 14 but declined by week 54, whereas concentrations remained stable in those with clinical remission (data not shown). These findings raise the possibility that elevated early tissue exposure may be associated with subsequent loss of response, although no distinct immunologic phenotype was identified to characterize this subgroup. Thus, while T cell immunophenotype at week 54 may reflect disease activity, it does not appear to serve as a predictive biomarker for loss of response. Further studies are warranted to explore whether early pharmacokinetic or immunologic signals can reliably identify patients at risk of losing response over time.

Consistent with the current study, a retrospective study in patients with UC treated with vedolizumab observed more CD4^+^ cells in colonic mucosal tissue from individuals who achieved remission than those without remission; high CD4^+^ cell infiltration was an independent predictor of response at 22 weeks [27]. Infiltration of CD4^+^ T cells may be an important predictive marker of response after 14 weeks of vedolizumab treatment. A single-cell transcriptomic study of colorectal tissue in patients with UC treated with vedolizumab revealed that T cells may use α4β7-independent intestinal trafficking pathways, and that myeloid dendritic cells are also affected by α4β7 blockade [28]. Although that study did not focus on mononuclear phagocytes such as fibroblasts, monocytes, macrophages, or mast cells, it identified T cell subsets that differed between responders and non-responders despite relatively subtle tissue-level changes. Our findings, particularly the differential proportions of CD161 ⁺ memory CD4 ⁺ T cells in inflamed tissue, are consistent with this observation and support the notion that T cell immunophenotypes may serve as important correlates of treatment response. However, our bulk immunophenotyping approach does not capture the full heterogeneity of the immune microenvironment, and future studies using single-cell resolution may help to further elucidate the cellular pathways underlying vedolizumab responsiveness.

Our study had several important strengths. Endoscopy results and colorectal tissue biopsy specimens were available from assessments at baseline (before treatment), week 14, and week 54. A limitation of our study is that it did not analyze innate immune or non-immune cells in the colorectal mucosa, and their contribution to response to vedolizumab could thus not be assessed. Future mechanistic studies are needed to elucidate further whether vedolizumab action includes mechanisms unrelated to lymphocyte trafficking. Mucosal biopsy tissue from colonoscopy contains microvessels, which limit its clear distinction from serum. The sample size is a key limitation that affected the statistical analyses that could be performed.

In conclusion, in this study in patients with UC, vedolizumab serum and tissue concentrations and α4β7 integrin saturation did not differ significantly between patients with and without remission at weeks 14 and 54. The effect of vedolizumab on the mucosal immune system may be qualitative rather than quantitative. Infiltration of CD4^+^ cells from blood at week 14 and CD161^+^ T cell proportions in tissue before treatment may be promising markers for predicting vedolizumab therapeutic efficacy. The relationship between pre-treatment CD161^+^ T cell proportions in tissue and vedolizumab efficacy has not been reported previously.

## Supporting information

S1 Fig Flow cytometry gating strategy. (PDF)

S2 Fig Complete Mayo score and biomarker (fecal calprotectin and soluble MAdCAM-1) levels. (PDF)

S1 Table Antibodies used for fluorescence-activated cell sorting analysis. (PDF)

S2 Table Immunophenotyping. (PDF)

S3 Table Non-parametric analysis of complete Mayo score by clinical remission status. (XLSX)

S4 Table Non-parametric analysis of fecal calprotectin and blood MAdCAM-1 by clinical remission status. (XLSX)

S5 Table Non-parametric analysis of vedolizumab concentrations in serum and colorectal tissue by clinical remission status. (XLSX)

S6 Table Non-parametric analysis of lymphocyte α4β7 integrin saturation in blood and colorectal tissue by clinical remission status. (XLSX)

S7 Table Non-parametric analysis of T cell immunophenotype proportions in blood and colorectal tissue by clinical remission status. (XLSX)

S8 Table Non-parametric analysis of T cell immunophenotype proportions in blood and colorectal tissue by response type (clinical remission and loss of clinical remission). (XLSX)

S1 File Protocol. (PDF)

S2 File Listing data. (XLSX)

S3 File CONSORT-2010-Checklist_TissuePK_final_09Jan26. (DOCX)
