# Supplementary material for: Association of tissue lymphocyte immunophenotype and clinical outcomes: A prospective study in patients with ulcerative colitis treated with vedolizumab
Source: PLoS One. 2026 Feb 3;21(2):e0340271. doi: 10.1371/journal.pone.0340271 (PMC12867234; doi:10.1371/journal.pone.0340271)

Fig S1. Flow cytometry gating strategy. A, area; FSC, forward scatter; H, height; SSC, side scatter, W, width.

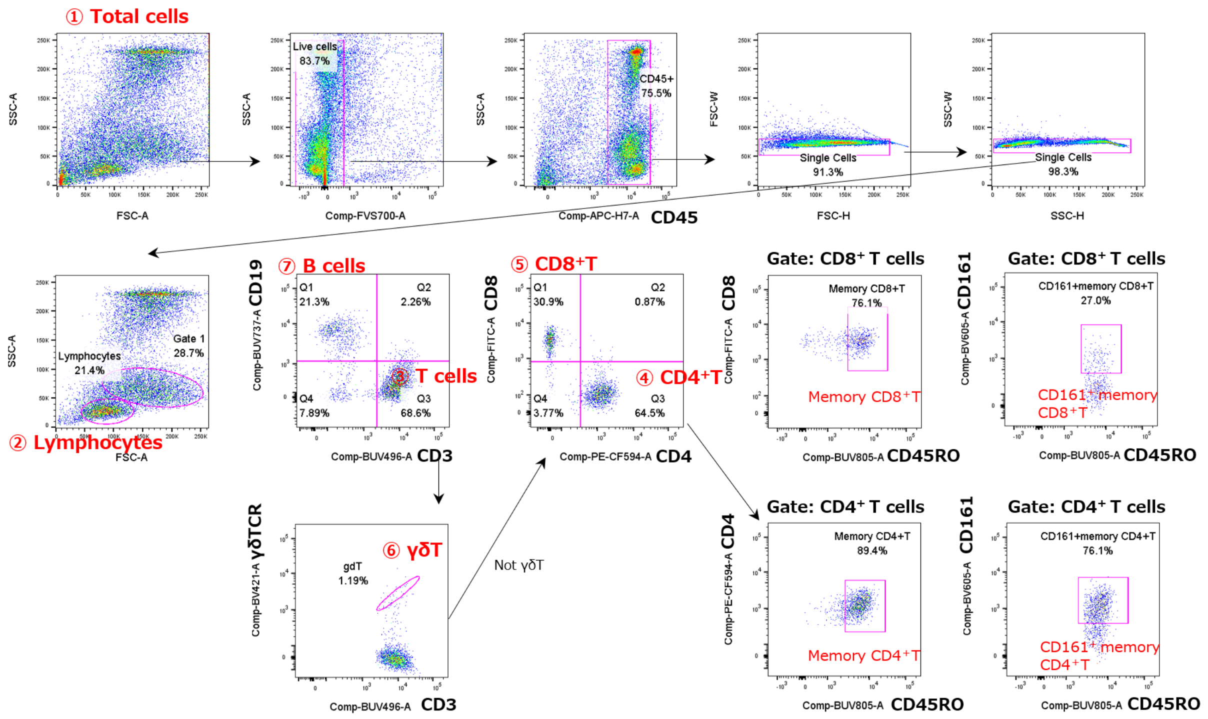

Supplement: S1 Fig — (PDF) [file pone.0340271.s001.pdf]
