# Supplementary material for: Association of tissue lymphocyte immunophenotype and clinical outcomes: A prospective study in patients with ulcerative colitis treated with vedolizumab
Source: PLoS One. 2026 Feb 3;21(2):e0340271. doi: 10.1371/journal.pone.0340271 (PMC12867234; doi:10.1371/journal.pone.0340271)

Fig S2. Complete Mayo score and biomarker (fecal calprotectin and soluble MAdCAM-1) levels. Complete Mayo score, and fecal calprotectin and soluble MAdCAM-1 levels at baseline, week 14, and week 54 according to week 54 clinical remission. Graphs are based on observed data; patients with missing data were excluded from analyses. Horizontal lines depict the Q1–Q3 range and median, whiskers represent the minimum and maximum values, and diamonds depict the mean. MAdCAM-1, Mucosal addressin cell adhesion molecule-1; Q, quartile.

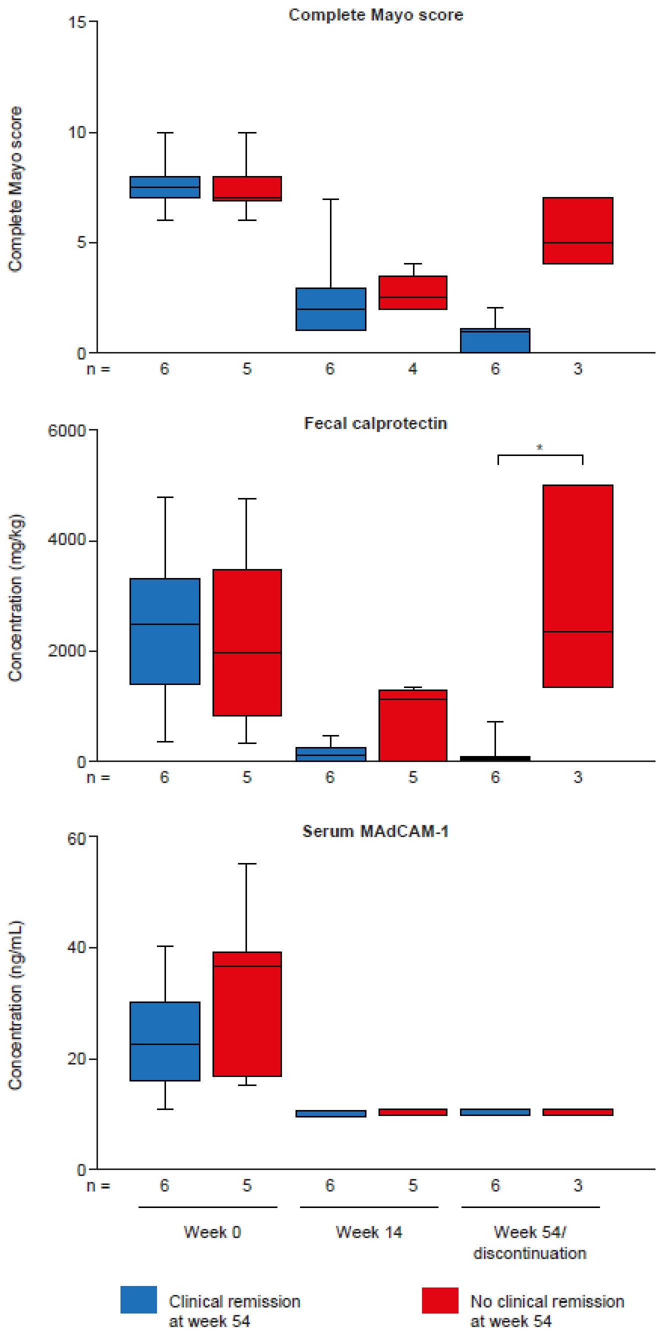

Supplement: S2 Fig — (PDF) [file pone.0340271.s002.pdf]
