# Supplementary material for: Association of tissue lymphocyte immunophenotype and clinical outcomes: A prospective study in patients with ulcerative colitis treated with vedolizumab
Source: PLoS One. 2026 Feb 3;21(2):e0340271. doi: 10.1371/journal.pone.0340271 (PMC12867234; doi:10.1371/journal.pone.0340271)
Supplement: S1 Table — (PDF) [file pone.0340271.s003.pdf]

Table S1. Antibodies used for fluorescence-activated cell sorting analysis.

**Marker**[a]	**Fluorophore**	**Clone**
CD45	APC-H7	2D1
CD3	BUV496	UCHT1
CD4	PE-CF594	L200
CD8	PE-Cy7	RPA-T8
CD45RO	BUV805	UCHL1
CD161	BV605	HP-3G10
CD25	APC	M-A251
CD127	BV711	HIL-7R-M21
γδTCR	BV421	11F2
CD19	BUV737	SJ25-C1
ACT-1[b]	PE[c]	

TCR, T cell receptor.

[a]Supplier: BD Biosciences.

[b]Biotinylated.

[c]Streptavidin-conjugated.
