# Supplementary material for: Association of tissue lymphocyte immunophenotype and clinical outcomes: A prospective study in patients with ulcerative colitis treated with vedolizumab
Source: PLoS One. 2026 Feb 3;21(2):e0340271. doi: 10.1371/journal.pone.0340271 (PMC12867234; doi:10.1371/journal.pone.0340271)
Supplement: S2 Table — (PDF) [file pone.0340271.s004.pdf]

Table S2. Immunophenotyping.

**Cells**	**Immunophenotype**	**Proportions**
T cells	$CD45^{+}CD3^{+}CD19^{-}$	–
$CD4^{+}$ T cells	$CD45^{+}CD3^{+}CD4^{+}CD8^{-}$	% T cells
Naïve $CD4^{+}$ T cells	$CD45^{+}CD3^{+}CD4^{+}CD8^{-}CD45RO^{-}$	% $CD4^{+}$ T cells
Memory $CD4^{+}$ T cells	$CD45^{+}CD3^{+}CD4^{+}CD8^{-}CD45RO^{+}$	% $CD4^{+}$ T cells
$CD161^{+}$ memory $CD4^{+}$ T cells	$CD45^{+}CD3^{+}CD4^{+}CD8^{-}CD45RO^{+}CD161^{+}$	% $CD4^{+}$ T cells
$CD8^{+}$ T cells	$CD45^{+}CD3^{+}CD4^{-}CD8^{+}$	% T cells
Naïve $CD8^{+}$ T cells	$CD45^{+}CD3^{+}CD4^{-}CD8^{+}CD45RO^{-}$	% $CD8^{+}$ T cells
Memory $CD8^{+}$ T cells	$CD45^{+}CD3^{+}CD4^{-}CD8^{+}CD45RO^{+}$	% $CD8^{+}$ T cells
$CD161^{+}$ memory $CD8^{+}$ T cells	$CD45^{+}CD3^{+}CD4^{-}CD8^{+}CD45RO^{+}CD161^{+}$	% $CD8^{+}$ T cells
T_{reg} cells	$CD45^{+}CD3^{+}CD4^{+}CD8^{-}CD25^{+}CD127^{low}$	% $CD4^{+}$ T cells
γδ T cells	$CD45^{+}CD3^{+}\gamma\delta TCR^{+}$	% T cells

T_{reg} cells, regulatory T cells.
