# Supplementary material for: Association of tissue lymphocyte immunophenotype and clinical outcomes: A prospective study in patients with ulcerative colitis treated with vedolizumab
Source: PLoS One. 2026 Feb 3;21(2):e0340271. doi: 10.1371/journal.pone.0340271 (PMC12867234; doi:10.1371/journal.pone.0340271)
Supplement: S1 File — (PDF) [file pone.0340271.s011.pdf]

PROTOCOL

A study to evaluate the relationship between colonic tissue and serum vedolizumab concentrations, α4β7 integrin receptor occupancy and efficacy in subjects with moderately to severely active ulcerative colitis and treated by vedolizumab IV

**Representative institution**	Asahikawa Medical University Hospital
**Secondary sponsor**	Takeda Pharmaceutical Company Limited
**Protocol number**	Vedolizumab-4026
**Version Number**	Version 2.0
**Study drug:**	Vedolizumab
**Creation date**	December 9, 2021

1.0 ADMINISTRATIVE INFORMATION AND PRINCIPLES OF CLINICAL STUDY

1.1 Principles of Clinical Study

This study is conducted with the highest respect for the individual participants in accordance with the protocol and the following requirements, and in accordance with ICH Guideline E6 for Good Clinical Practice.

- The ethical principles that have their origin in the Declaration of Helsinki
- Clinical Trials Act (No. 16 of 2017)
- All applicable laws and regulations, including, without limitation, data privacy laws and conflict of interest guidelines

1.2 Clinical Study Implementation Organization

This study is conducted in Japan with the following organization. Other study administrative organization are shown in the annexes.

Representative institution (institution of principal investigator):
Asahikawa Medical University Hospital

Secondary Sponsor:
Takeda Pharmaceutical Company Limited (Takeda)

Among the responsibilities for implementing this study that the principal investigator should assume, Takeda is responsible in cooperation with the principal investigator for the planning and the funding of the study.

The principal investigator or Takeda supervise the outsourcing contractor for this study by receiving a status report of the work performed by the outsourcing contractor based on the manual of procedures, provided separately and as guidance as necessary. If Takeda oversees the outsourcing contractor, Takeda reports the results to the principal investigator.

Expenses* required for the operation of this study are paid by Takeda.

*: Based on the “Consignment Service Contract,” expenses incurred for the services of the Office of Clinical Study, monitoring, registration/allocation center, and statistical

processing are paid to the contractor entrusted with services related to this study. Expenses agreed by the study site are paid to the site based on the "Research Expense Standard."

1.3 Study Period

Attachment 1

1.4 Contact Information on the Protocol

TABLE OF CONTENTS

1.0 ADMINISTRATIVE INFORMATION AND PRINCIPLES OF CLINICAL STUDY 3
1.1 Principles of Clinical Study 3
1.2 Clinical Study Implementation Organization 3
1.3 Study Period 4
1.4 Contact Information on the Protocol 4
2.0 STUDY SUMMARY 10
3.0 LIST OF ABBREVIATIONS 14
3.1 Study Definitions 14
4.0 INTRODUCTION 15
4.1 Background 15
4.2 Rationale for the Proposed Study 16
5.0 STUDY OBJECTIVES AND ENDPOINTS 17
5.1 Objectives 17
5.1.1 Primary Objectives 17
5.1.2 Secondary Objectives 17
5.1.3 Exploratory Objectives 17
5.2 Endpoints 17
5.2.1 Primary Endpoints 17
5.2.2 Secondary Endpoints 17
5.2.3 Exploratory endpoints 18
5.2.4 Safety Endpoint 19
6.0 STUDY DESIGN AND DESCRIPTION 20
6.1 Study Design 20
6.2 Justification for Study Design 21
6.3 Premature Termination of Study or Study Site 21
6.3.1 Criteria for Premature Termination of the Study 21
6.3.2 Criteria for Premature Termination of Study Sites 21

6.3.3 Procedures for Premature Termination or Suspension of the Study or the Participation of Study Sites 21
7.0 SELECTION AND DISCONTINUATION/WITHDRAWAL OF SUBJECTS 22
7.1 Inclusion Criteria 22
7.2 Exclusion Criteria 23
7.3 Excluded Medications 24
7.3.1 Justification for excluded medications 24
7.4 Instruction to the subject 24
7.5 Criteria for Discontinuation or Withdrawal of a Subject 25
7.6 Procedures for Discontinuation or Withdrawal of a Subjects 26
8.0 STUDY TREATMENT..27
8.1 Study Drug...27
8.1.1 Study drug 27
8.1.2 Dose and Regimen...27
9.0 STUDY PLAN 28
9.1 Study Procedures 28
9.1.1 Informed Consent Procedure 28
9.1.2 Demographics and Medication History..28
9.1.3 Physical Examination Procedure 29
9.1.4 Weight, Height and Body Mass Index 29
9.1.5 Documentation of Concomitant Drugs...29
9.1.6 Documentation of Concurrent Medical Conditions..........................30
9.1.7 Tissue Vedolizumab Concentration 30
9.1.8 Serum Vedolizumab Concentration 30
9.1.9 α4β7 Receptors Occupancy in Various Lymphocyte Populations in the Colonic Tissue and blood 31
9.1.10 The Patient Diary...31
9.1.11 Complete Mayo Score and Partial Mayo Score................................31
9.1.12 AVA/neutralizing AVA..33
9.1.13 Transcriptome Analysis of the Colonic Tissue.................................34

9.1.14 Immuno-phenotyping in the Colonic Tissue and blood 34
9.1.15 Biomarkers 34
9.1.16 Record of Subjects Who Are Withdrawn Before Start of Administration 34
9.2 Monitoring Subject Treatment Compliance 35
9.3 Schedule of Observations and Procedures 35
9.3.1 At Screening [VISIT 1] 35
9.3.2 Baseline [VISIT 2] 35
9.3.3 At Week 2 [VISIT 3] and Week 6 [VISIT 4] 36
9.3.4 At Week 14 [VISIT 5] 36
9.3.5 At Week 22 [VISIT 6] to 46 [VISIT 9] 37
9.3.6 At the End of Study [VISIT 10] or at the Early Termination 37
10.0 ADVERSE EVENTS 39
10.1 Definitions 39
10.1.1 PTEs 39
10.1.2 AEs39
10.1.3 Additional Points to Consider for PTEs and AEs 39
10.1.4 SAEs 42
10.1.5 AEs of particular interest 42
10.1.6 Intensity of PTEs and AEs 42
10.1.7 Causality of AEs 42
10.1.8 Relationship to Study Procedures 43
10.1.9 Start Date 43
10.1.10Stop Date 44
10.1.11Frequency 44
10.1.12Action Concerning Study Drug 44
10.1.13Outcome 44
10.2 Procedures 45
10.2.1 Collection of AEs 45
*10.2.1.3*AEs of particular interest 46
10.2.2 Reporting of SAEs 46

10.2.3 Reporting of Non-serious AEs 47
10.2.4 Reporting of Special Situation 47
10.2.5 Abnormal liver function tests 47
10.2.6 Follow-up of AEs 47
10.2.7 Reporting of Additional Information on Adverse Events 48
10.3 Reporting of Disease or the Like 48
10.3.1 Reporting of Disease or the Like to CRB 48
10.3.2 Reporting of Safety Data to Regulatory Authority 48
11.0 STUDY-SPECIFIC COMMITTEES 49
12.0 DATA HANDLING AND RECORDKEEPING 50
12.1 CRFs (Electronic) 50
12.2 Record Retention 51
13.0 STATISTICAL METHODS 52
13.1 Statistical and Analytical Plans 52
13.1.1 Analysis Sets 52
13.1.2 Analysis of Demographics and Other Baseline Characteristics 52
13.1.3 Pharmacokinetics and Efficacy Analysis 52
13.1.4 Safety Analysis 55
13.2 Interim Analysis and Criteria for Early Termination 55
13.3 Determination of Sample Size 55
14.0 QUALITY CONTROL AND QUALITY ASSURANCE 56
14.1 Study-Site Monitoring Visits 56
14.2 Protocol Deviation 56
14.3 Quality Assurance Audits and Regulatory Agency Inspections 57
15.0 ETHICAL ASPECT OF THE STUDY 58
15.1 CRB Approval 58
15.2 Subject Information, and Informed Consent 58
15.3 Procedures for Study Plan Revision 60
15.4 Subject Confidentiality 60
15.5 Conflict of Interests 60
15.6 Financial Burden 61

15.7 Benefits and Inconveniences to Subjects.....61
15.7.1 Benefits to Subjects.....61
15.7.2 Inconveniences to Subjects.....61
15.7.3 Ethical Considerations for Study Design.....62
15.8 Attribution of Study Results.....62
15.9 Publication, Disclosure, and Clinical Trial Registration Policy.....62
15.9.1 Publication and Disclosure.....62
15.9.2 Clinical Study Registration.....63
15.9.3 Clinical Study Results Disclosure.....63
15.10 Insurance and Compensation for Injury.....63
16.0 REFERENCES.....65

LIST OF IN-TEXT TABLES

Table 9.a Mayo Scoring System for the Assessment of UC Activity.....31
Table 9.b Biomarkers.....34

LIST OF APPENDICES

Appendix A Schedule of Study Procedures.....67
Appendix B Special situation to be reported to Takeda.....68
Appendix C Responsibilities of the investigator.....69

2.0 STUDY SUMMARY

Study Drug: Vedolizumab (Vedolizumab IV 300 mg)

Title of Protocol:

A study to evaluate the relationship between the colonic tissue and serum vedolizumab concentrations, α4β7 integrin receptor occupancy and efficacy in subjects with moderately to severely active ulcerative colitis and treated by vedolizumab IV

Study Number: Vedolizumab-4026

Study Design:

This is an open label, single-arm, multicenter study to evaluate the relationship between colonic tissue and serum vedolizumab concentrations, α4β7 integrin receptor occupancy and efficacy in subjects with moderately to severely active ulcerative colitis (UC) and treated by vedolizumab 300 mg intravenous (IV) infusion over a 54 week-treatment period. The intention is to better characterize the differences in efficacy of vedolizumab observed between biologic-naïve and -exposed patients. Moderately to severely active UC is defined as a complete Mayo score of 6 to 12 with endoscopic subscore of ≥2. Efficacy is evaluated by the proportion of subjects with clinical remission and the proportion of subjects with mucosal healing.

Primary Objectives:

- To assess the relationship between the clinical outcomes and concentrations of vedolizumab in the colonic tissue (inflamed / non inflamed) and in the serum

Secondary Objectives:

- To evaluate the relationship between α4β7 integrin receptor occupancy and the clinical outcomes
- To assess the relationship between the concentrations of vedolizumab in the colonic tissue and in the serum
- To determine the α4β7 receptors occupancy in various lymphocyte populations in the colonic tissue and in the blood
- To assess the relationship between the concentrations of vedolizumab in the colonic tissue and in the serum and the α4β7 receptors occupancy, and clinical outcomes in subjects with or without previous exposure to TNFα antagonist
- To evaluate the effect of anti-vedolizumab antibodies (AVA) and neutralizing antibodies on the therapeutic effect

Exploratory Objective:

- To investigate the relationship between the concentrations of vedolizumab in the colonic tissue and in the serum and various biomarkers such as sMAdCAM-1, CRP and fecal calprotectin

• To assess the relationship between the colonic tissue transcriptome and clinical outcomes<br>• To assess the impact of vedolizumab on various immune-cell populations in the colonic tissue and blood	
**Subject Population:** Subjects with UC	
**Number of Subjects:**<br>30 subjects<br>Approximately half of the enrolled subjects are TNFα antagonist naïve.	**Number of Sites:**<br>Approximately 3 sites
**Dose Level:**<br>Vedolizumab 300 mg is administered at Weeks 0, 2, 6 and every 8 weeks thereafter.	**Route of Administration:**<br>Intravenous infusion
**Duration of Treatment:**<br>46 weeks	**Period of Evaluation:**<br>54 weeks
**Main Criteria for Inclusion:**<br>• Subjects who have had a diagnosis of UC for at least 3 months prior to enrollment<br>• Subjects with complete Mayo score of 6-12 and endoscopic subscore of ≥2 within 10 days before the initial administration of vedolizumab<br>• Subjects who met the following treatment failure criteria with at least one of the following agents:<br>Corticosteroids<br>• Resistance<br>• Dependence<br>• Intolerance<br>Immunomodulators (azathioprine, 6-mercaptopurine or methotrexate)<br>• Refractory<br>• Intolerance<br>TNFα antagonist<br>• Inadequate response<br>• Loss of response<br>• Intolerance<br>• Subject aged 20-80 years at informed consent.	
**Main Criteria for Exclusion:**<br>• Subjects who had extensive colonic resection, subtotal or total colectomy<br>• Subjects who are classified as proctitis UC (inflammation is limited to the rectum)	

- Subjects who had ileostomy, colostomy, or symptomatic intestinal stricture
- Subject who had received any of the following biologic retroactively from the planned first dose of vedolizumab within the following period: Infliximab 8 weeks, adalimumab 2 weeks, golimumab 4 weeks, JAK inhibitor 1 week, ustekinumab 8 weeks
- Subjects who had prior exposure to vedolizumab, natalizumab, efalizumab or rituximab (any time before)
- Subjects who had any evidence of an active infection within 1 month prior to the first administration of vedolizumab
- Subject with a history of hypersensitivity or allergies to vedolizumab or its components (subjects with contraindication in the vedolizumab package insert)
- Subject having a concurrent or a history of malignancy
- Patients who have been determined to be inappropriate as subjects in the study by the investigator

Endpoints and Assessments:

Primary endpoints

- Concentrations of vedolizumab in the colonic tissue and in the serum, and clinical remission (complete Mayo score ≤ 2 and all sub-scores ≤ 1) rate at Week 54

Secondary endpoints

- Concentrations of vedolizumab in the colonic tissue and in the serum, and clinical remission rate at Week 14
- Correlation between the concentrations of vedolizumab in the colonic tissue and in the serum, and mucosal healing (endoscopy Mayo sub-score ≤ 1) at Week 14 and 54
- Correlation between the α4β7 integrin receptor occupancy and clinical outcomes (clinical remission and mucosal healing) at Week 14 and 54
- Correlation between the colonic tissue and serum concentrations of vedolizumab and α4β7 integrin receptor occupancy at Week 14 and 54
- Concentrations of vedolizumab in the colonic tissue and in the serum at Week 14 and 54
- α4β7 receptors occupancy in various lymphocyte populations in the colonic tissue and in the blood at Week 14 and 54
- Proportion of subjects achieving clinical remission and mucosal healing at Week 14 and 54
- Correlation between the concentrations of vedolizumab in the colonic tissue and in the serum, α4β7 integrin receptor occupancy and clinical outcomes in subjects with or without previous exposure to TNFα antagonist at Week 14 and 54
- Concentrations of vedolizumab in the colonic tissue and in the serum in subjects with or without

previous exposure to TNFα antagonist at Week 14 and 54<br>• Proportion of subjects achieving clinical remission and mucosal healing in subjects with or without previous exposure to TNFα antagonist at Week 14 and 54<br>• Correlation between anti-vedolizumab antibody (AVA) and neutralizing AVA and clinical outcomes at Week 14 and 54<br>• Proportion of subjects positive for AVA and neutralizing AVA at Baseline, Week 14 and 54<br>**Exploratory endpoints**<br>• Biomarkers concentrations such as sMAdCAM-1, CRP and fecal calprotectin, at Week 0 (baseline), 14 and 54<br>• Transcriptome analysis of the colonic tissue at Week 0 (baseline), 14 and 54<br>• Correlation between the colonic tissue transcriptome and clinical outcomes at Week 14 and 54<br>• Correlation between the concentrations of vedolizumab in the colonic tissue and in the serum and immuno-phenotyping of various immune-cell populations at Week 14 and 54<br>• Immuno-phenotyping of various immune-cell populations at Week 0 (baseline), 14 and 54<br>**Safety endpoint**<br>• Adverse events
**Statistical Considerations:**<br>[Vedolizumab concentrations in the colonic tissue/serum, α4β7 integrin receptor occupancy and clinical outcomes]<br>The remission and mucosal healing rates will be calculated for each category of vedolizumab concentration in colon tissue and serum and α4β7 integrin receptor occupancy at each evaluation time point. Summary statistics of tissue and serum vedolizumab concentrations and α4β7 receptor saturation will also be calculated by remission and mucosal healing.<br>[Safety]<br>Incidence of adverse events is summarized.
**Sample Size Justification:**<br>A sample size of 30 subjects was defined based on feasibility reasons.

3.0 LIST OF ABBREVIATIONS

AE	adverse event
ALT	alanine aminotransferase
AST	aspartate aminotransferase
AVA	anti-vedolizumab antibody
CRB	certified review board
CRF	case report form
CRP	C-reactive protein
GCP	Good Clinical Practice
ICH	International Conference on Harmonisation
INR	international normalized ratio
IV	Intravenous
MedDRA	Medical Dictionary for Regulatory Activities
PK	pharmacokinetics
PTE	pretreatment event
TNF	tumor necrosis factor
UC	ulcerative colitis

3.1 Study Definitions

The definitions in the study are provided below;

Terms	Definitions
Clinical remission	Complete Mayo score ≤ 2 and all sub-scores ≤ 1
Mucosal healing	Endoscopy Mayo sub-score ≤ 1

4.0 INTRODUCTION

4.1 Background

Ulcerative colitis (UC) is an inflammatory bowel disease that repeatedly recurs and remits. Clinical manifestations include diarrhea, typically bloody, as well as abdominal pain, fecal urgency, and incontinence. Systemic features such as fever, weight loss, malaise, and fatigue are indicators of more extensive disease. UC is characterized by superficial, continuous mucosal inflammation and ulcers that are restricted to the colon.

While the etiology remains unknown, recent studies have shown that a lower immune resistance to intraluminal antigens including enterobacteria and abnormal cytokines may be a contributing factor. A curative treatment has not been established yet; therefore, the medical approach intends basically to promptly induce clinical remission and to maintain it for the longest (remission maintenance) with pharmacotherapy. The guidance of pharmacotherapy is provided in the Revised Guideline or Guidance [1] issued by Research Group for Intractable Inflammatory Bowel Disease Designated as Specified Disease by the Ministry of Health, Labour and Welfare (MHLW) of Japan, however treatment should be detailed based on disease types or severities.

Vedolizumab is a recombinant humanized immunoglobulin G1 monoclonal antibody that specifically binds to and inhibits α4β7 integrin, a cell membrane protein of lymphocytes, from binding to its principal ligand of mucosal addressin cell adhesion molecule-1, leading to suppression of lymphocytes to migrate toward the intestinal mucosa and the gut-related lymphoid tissue [2, 9]. Vedolizumab has been approved in Japan, the United States, European Union, and multiple other countries as the indication for the treatment of adult patients with moderately to severely active UC and Crohn’s disease.

In the phase 3 studies conducted in Japan and outside Japan, the efficacy and the safety of vedolizumab were initially evaluated versus placebo during both the induction and the maintenance phases in moderate to severe UC patients who had failed with the existing pharmacotherapy including tumor necrosis factor (TNF) α antagonists [3, 4]. In a recent study, vedolizumab has demonstrated a superior clinical benefit to adalimumab in moderate to severe UC patients – VARSITY [5]. A post hoc analysis of GEMINI 1 has suggested that higher trough serum concentrations of vedolizumab could favor higher rates of clinical remission and clinical response in patients with UC [3, 6].

4.2 Rationale for the Proposed Study

Yarur AJ et al. have reported that some patients with high serum TNFα antagonist levels were nevertheless showing high inflammation and significant clinical symptoms. Hypothesis was that tissue levels of TNFα antagonist were insufficient to neutralize local TNF production [7, 10].

The tissue concentration of vedolizumab may affect the clinical efficacy of vedolizumab, but the relationship between the tissue concentration of vedolizumab and its efficacy has never been investigated.

The proposed study assesses the relationship between the colonic tissue and serum vedolizumab concentrations, α4β7 integrin receptor occupancy and efficacy in subjects with moderately to severely active ulcerative colitis and treated by vedolizumab IV with the intention to better characterize the differences of vedolizumab efficacy observed between biologic-naïve and -exposed patients.

5.0 STUDY OBJECTIVES AND ENDPOINTS

5.1 Objectives

5.1.1 Primary Objectives

- To assess the relationship between the clinical outcomes and concentrations of vedolizumab in the colonic tissue (inflamed / non inflamed) and in the serum

5.1.2 Secondary Objectives

- To evaluate the relationship between α4β7 integrin receptor occupancy and the clinical outcomes
- To assess the relationship between the concentrations of vedolizumab in the colonic tissue and in the serum
- To determine the α4β7 receptors occupancy in various lymphocyte populations in the colonic tissue and in the blood
- To assess the relationship between the concentrations of vedolizumab in the colonic tissue and in the serum and the α4β7 receptors occupancy, and clinical outcomes in subjects with or without previous exposure to TNFα antagonist
- To evaluate the effect of AVA and neutralizing antibodies on the therapeutic effect

5.1.3 Exploratory Objectives

- To investigate the relationship between the concentrations of vedolizumab in the colonic tissue and in the serum and various biomarkers such as sMAdCAM-1, CRP and fecal calprotectin
- To assess the relationship between the colonic tissue transcriptome and clinical outcomes
- To assess the impact of vedolizumab on various immune-cell populations in the colonic tissue and blood

5.2 Endpoints

5.2.1 Primary Endpoints

- Correlation between the concentrations of vedolizumab in the colonic tissue and in the serum, and clinical remission (complete Mayo score ≤ 2 and all sub-scores ≤ 1) at Week 54

5.2.2 Secondary Endpoints

- Concentrations of vedolizumab in the colonic tissue and in the serum, and clinical

remission rate at Week 14

- Correlation between the concentrations of vedolizumab in the colonic tissue and in the serum, and mucosal healing at Week 14 and 54
- Correlation between the α4β7 integrin receptor occupancy and clinical outcomes (clinical remission and mucosal healing) at Week 14 and 54
- Correlation between the colonic tissue and serum concentrations of vedolizumab and α4β7 integrin receptor occupancy at Week 14 and 54
- Concentrations of vedolizumab in the colonic tissue and in the serum at Week 14 and 54
- α4β7 receptors occupancy in various lymphocyte populations in the colonic tissue and in the blood at Week 14 and 54
- Proportion of subjects achieving clinical remission and mucosal healing at Week 14 and 54
- Correlation between the concentrations of vedolizumab in the colonic tissue and in the serum, α4β7 integrin receptor occupancy and clinical outcomes in subjects with or without previous exposure to TNFα antagonist at Week 14 and 54
- Concentrations of vedolizumab in the colonic tissue and in the serum in subjects with or without previous exposure to TNFα antagonist at Week 14 and 54
- Proportion of subjects achieving clinical remission and mucosal healing in subjects with or without previous exposure to TNFα antagonist at Week 14 and 54
- Correlation between AVA and neutralizing AVA and clinical outcomes at Week 14 and 54
- Proportion of subjects positive for AVA and neutralizing AVA at Baseline, Week 14 and 54

5.2.3 Exploratory endpoints

- Biomarkers concentrations such as sMAdCAM-1, CRP and fecal calprotectin, at Week 0 (baseline), 14 and 54
- Transcriptome analysis of the colonic tissue at Week 0 (baseline), 14 and 54
- Correlation between the colonic tissue transcriptome and clinical outcomes at Week 14 and 54
- Correlation between the concentrations of vedolizumab in the colonic tissue and in the serum and immuno-phenotyping of various immune-cell populations at Week 14 and 54

- Immuno-phenotyping of various immune-cell populations at Week 0 (baseline), 14 and 54

5.2.4 Safety Endpoint

- Adverse events

6.0 STUDY DESIGN AND DESCRIPTION

6.1 Study Design

<Study design>

This is an open label, single-arm, multicenter study to evaluate the relationship between the colonic tissue and serum concentrations of vedolizumab, α4β7 integrin receptor occupancy and efficacy in subjects with moderately to severely active UC and treated by vedolizumab 300 mg IV infusion over a 54 week-treatment period. The intention is to better characterize the differences of vedolizumab efficacy observed between biologic-naïve and -exposed patients. Moderately to severely active UC is defined as a complete Mayo score of 6 to 12 with endoscopic subscore of ≥2. The efficacy is evaluated by the proportion of subjects with clinical remission and the proportion of subjects with mucosal healing.

< Period of Evaluation >

54 weeks

<Treatment>

Vedolizumab 300 mg is administered at Week 0, 2, 6 and every 8 weeks thereafter. Treatment duration is 46 weeks.

<Number of Subjects>

30 subjects

Approximately half of the subjects are those who have not been previously treated with an anti-TNFα antibody product. The study office will check the inclusion status of research subjects using electronic case report forms, etc., and share this information with the principal investigators and sub-investigators at each institution so that approximately half of the research subjects will have no history of prior treatment with anti-TNFα antibody products.

<Number of visits>

10 visits

<Number of study sites>

Approximately 3 sites

A schedule of assessments is listed in Appendix A.

6.2 Justification for Study Design

(1) Justification for study design

Since the objective of this study is to assess the relationship between the concentrations of vedolizumab in the colonic tissue and in the serum, $\alpha 4\beta 7$ integrin receptor occupancy and efficacy, it is designed as a single arm and open-label study.

(2) Justification for dose and administration method

Vedolizumab is administered in accordance with the package insert.

(3) Justification for the planned number of subjects

Refer to Section 13.3.

6.3 Premature Termination of Study or Study Site

6.3.1 Criteria for Premature Termination of the Study

The principal investigator or Takeda should immediately discontinue the study if 1 or more of the following criteria are satisfied:

- New information or other evaluation regarding the safety or efficacy of the study drug that indicates a change in the known risk/benefit profile for the product, such that the risk/benefit is no longer acceptable for subjects participating in the study.
- Significant violation of Clinical Trials Act that compromises subject safety.

6.3.2 Criteria for Premature Termination of Study Sites

A study site may be terminated prematurely if the site (including the investigator) is found in significant violation of the Clinical Trials Act, protocol, or contractual agreement, is unable to ensure adequate performance of the study, or as otherwise permitted by the contractual agreement.

6.3.3 Procedures for Premature Termination or Suspension of the Study or the Participation of Study Sites

In the event that the principal investigator, Takeda, Certified Review Board (CRB) or regulatory authority elect to terminate or suspend the study or the participation of a study site, a study-specific procedure for early termination or suspension will be provided by the principal investigator or Takeda; the procedure will be followed by any of the study sites during the course of termination or study suspension, if needed.

7.0 SELECTION AND DISCONTINUATION/WITHDRAWAL OF SUBJECTS

All entry criteria, including test results, need to be confirmed prior to first dose of the study drug.

7.1 Inclusion Criteria

Subject eligibility is determined according to the following criteria prior to entry into the study:

1. Subject who is capable of understanding and complying with protocol requirements in the opinion of the investigator.
2. Subject who signs and dates a written, informed consent form prior to the initiation of any study procedures.
3. Subjects who have had a diagnosis of moderate to severe UC for at least 3 months prior to enrollment
4. Subjects with complete Mayo score of 6-12 and endoscopic subscore of ≥2 within 10 days before the initial administration of vedolizumab
5. Subjects who met the following treatment failure criteria with at least one of the following agents:
   - Corticosteroids
     - Resistance: subjects whose response was inadequate after treatment of ≥40 mg/day for ≥1 week[1] (oral or intravenous [IV]) or 30 to 40 mg/day for ≥2 weeks (oral or IV) or topical therapy for ≥2 weeks in case of distal UC.
     - Dependence: subjects for which it was difficult to reduce the dosage to <10 mg/day due to recurrence during gradual dose reduction (oral or IV) or for which it was difficult to discontinue topical therapy.
     - Intolerance: subjects who were unable to receive continuous treatment due to adverse reactions (e.g., Cushing's syndrome, osteopenia/osteoporosis, hyperglycemia, insomnia, infection).
   - Immunomodulators (azathioprine [AZA] or 6-mercaptopurine [6-MP])
     - Refractory: subjects whose response was inadequate after treatment for ≥12 weeks.

- Intolerance: subjects who were unable to receive continuous treatment due to adverse reactions (e.g., nausea/vomiting, abdominal pain, pancreatitis, liver function test abnormalities, lymphopenia, thiopurine S-methyltransferase genetic mutation, infection).

- TNFα antagonist
  - Inadequate response: subjects whose response was inadequate after the induction therapy in the dosage described in the package insert.
  - Loss of response: subjects who had recurrence during the scheduled maintenance therapy after achievement of clinical response (those who withdrew for other reasons than relapse is not applicable here).
  - Intolerance: subjects who were unable to receive continuous treatment due to adverse reactions (e.g., infusion-related reaction, demyelination, congestive heart failure, infection).

6. Subject aged 20-80 years at informed consent.

[Justification of inclusion criteria]

These were set to include the subjects suitable for assessment of this study.

7.2 Exclusion Criteria

Any subject who meets any of the following criteria will not qualify for entry into the study:

1. Subjects who had extensive colonic resection, subtotal or total colectomy
2. Subjects who are classified as proctitis UC (inflammation is limited to the rectum)
3. Subjects who had ileostomy, colostomy, or symptomatic intestinal stricture
4. Subjects who had received any of the following biologic within the designated period before the initial administration of vedolizumab: infliximab (8 weeks before), adalimumab (2 weeks before), golimumab (4weeks before), JAK inhibitor (1 week before), ustekinumab (8 weeks before)
5. Subjects who had prior exposure to vedolizumab, natalizumab, efalizumab or rituximab (any time before)
6. Subjects who had any evidence of an active infection within 1 month prior to the first administration of vedolizumab
7. Subject with a history of hypersensitivity or allergies to vedolizumab or its components (subjects with contraindication in the vedolizumab package insert)
8. Subject having a concurrent or a history of malignancy

9. Patients who have been determined to be inappropriate as subjects in the study by the investigator.

[Justification for exclusion criteria]

1-5 These exclusion criteria were set because they might affect the evaluation of the efficacy.

6-9 These exclusion criteria were set to appropriately consider the safety of the subjects.

7.3 Excluded Medications

The following drugs should not be used concomitantly with the study drug during the study:

1. TNFα antagonist
2. JAK inhibitors
3. Ustekinumab

7.3.1 Justification for excluded medications

This exclusion criterion was set because it might affect the evaluation of the efficacy.

7.4 Instruction to the subject

1. To adhere the visit date and to receive medical examinations and prescribed test. To contact the investigator or research collaborator as soon as possible in case the subject cannot visit hospital on scheduled date.
2. To record the patient diary throughout the period from the start of administration of the study drug to the last dose of study drug and to bring it with each visit.
3. To report promptly to the investigator or research collaborator by telephone, etc., and to ask for instructions, in case symptoms worsen on a date other than the scheduled visit date.
4. To report the contents, onset date, degree, outcome, and outcome date of the subjective symptoms / objective findings to the investigator at the visit.
5. To report the details of treatment to the investigator in case the subject visits another medical institution from the informed consent until the end (VISIT 10) or at the time of discontinuation.

6. Not to violently eat and drink, to avoid extreme change the content of meals, to avoid exercise excessively or act during the period from informed consent until the end (VISIT 10) or at the time of discontinuation. Careful instruction is needed in case the symptoms change during the study period leading to change of the meal content
7. Not to donate blood from the informed consent until the end (VISIT 10) or at the time of discontinuation.6 months after the last dose of study drug.
8. To immediately report to the investigator when the subject feels an infusion reaction (rash, pruritus, flushing, urticaria, etc.)

7.5 Criteria for Discontinuation or Withdrawal of a Subject

The primary reason for discontinuation or withdrawal of the subject from the study or study drug should be recorded in the case report form (CRF) using the following categories. For subjects who withdraw from the study before administration, refer to Section 9.1.16.

1. **Adverse event**
   The subject has experienced an adverse event (AE) or a pretreatment event (PTE) that requires early termination because continued participation imposes an unacceptable risk to the subject's health, or the subject is unwilling to continue because of the AE. For abnormal liver function tests, discontinuation should be considered according to the following criteria.

   ・ Liver Function Test (LFT) Abnormalities.

   - Study drug should be discontinued immediately with appropriate clinical follow-up (including repeat laboratory tests, until a subject's laboratory profile has returned to normal/baseline status), if the following circumstances occur at any time during study drug treatment:
     alanine aminotransferase (ALT) or aspartate aminotransferase (AST) >8 × upper limit of normal (ULN), or
   - ALT or AST >5 × ULN and persists for more than 2 weeks, or
   - ALT or AST >3 × ULN in conjunction with elevated total bilirubin >2 × ULN or international normalized ratio (INR) >1.5, or
   - ALT or AST >3 × ULN with appearance of fatigue, nausea, vomiting, right upper quadrant pain or tenderness, fever, rash and/or eosinophilia (>5%).

2. **Significant protocol deviation**
   The discovery after the first dose of study drug that the subject failed to meet protocol entry criteria or did not adhere to protocol requirements, and continued participation poses an unacceptable risk to the subject's health.
3. **Lost to follow-up**
   The subject did not return to the clinic and attempts to contact the subject are unsuccessful. Attempts to contact the subject must be documented in the subject's source documents.
4. **Voluntary withdrawal**
   The subject wishes to withdraw from the study. The reason for withdrawal, if provided, should be recorded in the (e)CRF.

   Note: All attempts should be made to determine the underlying reason for the withdrawal and, where possible, the primary underlying reason should be recorded (i.e., withdrawal due to an AE should not be recorded in the "voluntary withdrawal" category).
5. **Study termination**
   The principal investigator, Takeda or CRB terminates the study.
6. **Lack of efficacy**
   The investigator has determined that the subject is not benefiting from study treatment; and, continued participation would pose an unacceptable risk to the subject.
7. **Others**

   The investigator determined to terminate the study for other reasons.

   Note: The specific reasons should be recorded on the (e)CRF.

7.6 Procedures for Discontinuation or Withdrawal of a Subjects

The investigator may discontinue a subject's study participation at any time during the study when the subject meets the study termination criteria described in Section 7.5. In addition, a subject may discontinue his or her participation without giving a reason at any time during the study. Should a subject's participation be discontinued, the primary criterion for termination must be recorded by the investigator. In addition, efforts should be made to perform all procedures scheduled for the Early Termination Visit.

8.0 STUDY TREATMENT

This section indicates the treatment regimen of this study. Marketed product in each study site is used. See the latest package insert for details and handling of the drug.

8.1 Study Drug

8.1.1 Study drug

Vedolizumab IV 300 mg

8.1.2 Dose and Regimen

Vedolizumab IV 300 mg is administered by intravenous infusion at Week 0, 2, 6 as induction therapy and every 8 weeks thereafter as maintenance therapy. Product preparation and infusion should be performed according to the current package insert.

9.0 STUDY PLAN

9.1 Study Procedures

The following sections describe the study procedures and data to be collected. For each procedure, subjects are to be assessed by the same investigator or site personnel whenever possible. The Schedule of Study Procedures is in Appendix A

9.1.1 Informed Consent Procedure

The requirements of the informed consent are described in Section 15.2.

Informed consent must be obtained prior to the subject entering into the study, and before any protocol-directed procedures are performed.

A unique subject identification number (subject number) is assigned to each subject at the time that informed consent is obtained; this subject number is used throughout the study.

9.1.2 Demographics and Medication History

Demographic information to be obtained includes date of birth (or age at the time of informed consent), sex, smoking status, and drinking history. In addition, the following UC-related items are obtained.

- Date primary disease diagnosed.
- The following item as defined in the Revised Diagnostic Criteria for UC issued by "Research Group for Intractable Inflammatory Bowel Disease" Designated as Specified Disease by the MHLW of Japan (2012).
  - Disease classification by extent of the UC lesion (total colitis or left-sided colitis).
- History of treatment failure with the following drugs.
  - Corticosteroids (resistance, dependence, or intolerance).
  - Immunomodulators (azathioprine, or 6-mercaptopurine) (refractory or intolerance).
  - TNFα antagonists* (inadequate response, loss of response, or intolerance).

  * For inadequate response and loss of response, the name and the first and last administration dates of the drug are recorded as well. For intolerance, the name of the drug and the side-effect leading to discontinuation are recorded as well.

Medical history to be obtained included any clinically significant conditions or diseases (e.g., extraintestinal manifestations, pouchitis, infectious enteritis, toxic megacolon, stenosis, and fistula) that had resolved within 1 year before signing of informed consent and are considered to be relevant to the study evaluation in the opinion of the investigator. In addition, any previous malignancies and enterectomy for UC are also obtained. Ongoing conditions or diseases are considered concurrent medical conditions.

Medication history to be obtained included the followings:

- Biologics for UC treatment discontinued before signing of informed consent.
- Corticosteroids and immunomodulators for UC treatment discontinued within 4 weeks before signing of informed consent.

Of note, agents applied for procedures including the endoscopy are not included in the medication history and not documented in the (e)CRF.

9.1.3 Physical Examination Procedure

All subsequent physical examinations should assess clinically significant changes from the assessment prior to first dose examination. During infusion and 1 hour after infusion of the study drug, infusion reactions (e.g., rash, pruritus, flushing, or urticaria) are observed.

9.1.4 Weight, Height and Body Mass Index

Weight and height are measured. Body Mass Index (BMI) is calculated from the following formula during statistical analysis.

Metric: BMI = weight (kg) / height $(m)^2$

Height is recorded in centimeters without decimal places. Weight is collected in kilograms with 1 decimal place. BMI should be report to 1 decimal place by rounding.

9.1.5 Documentation of Concomitant Drugs

Concomitant medication is any drug given in addition to the study medication. These are prescribed by a physician or obtained by the subject over the counter. At each study visit, subjects are asked whether they had taken any drug other than the study drug (used from signing of informed consent through the end-of-study (VISIT 10)), and all drugs including vitamin supplements, OTC drugs, and oral herbal preparations, had to be recorded in the (e)CRF. In addition, for concomitant drugs for UC treatment, the class of the concomitant drug (5-aminosalicylic acid, corticosteroids,

immunomodulators, or others), and its applicability to rescue medications is recorded in the (e)CRF.

Concomitant treatments are defined as any treatments for UC other than pharmacotherapy, including enterectomy and other surgical treatments (e.g., abscess drainage or the Seton method for the anal lesion). All information on concomitant treatments that had been conducted during the period from acquisition of the informed consent until the end-of-study examination is obtained. Additionally, applicability of the concomitant treatments to rescue medications is recorded in the (e)CRF.

Of note, agents applied for procedures including endoscopy are not considered as concomitant drugs and are also not documented in the (e)CRF.

9.1.6 Documentation of Concurrent Medical Conditions

Concurrent medical conditions are those significant ongoing conditions or diseases that are present when the patient signs the informed consent. This includes clinically significant laboratory, electrocardiogram (ECG), or physical examination abnormalities noted at the screening/baseline examination. The condition (i.e., diagnosis) is described. In addition, UC-related extraintestinal manifestations are recorded in the (e)CRF.

9.1.7 Tissue Vedolizumab Concentration

Tissue samples are obtained with biopsy forceps during colonoscopy from the non-inflamed and inflamed tissue in the colon. Tissue samples are homogenized in the buffer and centrifuged at 3000rpm at 4C for 5min. Then the supernatants are collected and cryopreserved in a preset freezer at a temperature of ≤-20°C. The detailed procedure is specified in a separate manual. Serum Vedolizumab concentration is determined by means of Enzyme-Linked Immunosorbent Assay at an outside laboratory.

9.1.8 Serum Vedolizumab Concentration

For measurement of serum vedolizumab concentration, 3 mL of the venous blood is collected. At the visit for study drug administration, the blood is sampled immediately before administration, where possible. The dates of the blood sampling are recorded in the (e)CRF.

The collected blood is left standing at room temperature for approximately 30 minutes and centrifuged at 3,000 rpm at 4°C for 10 minutes. The obtained serum is cryopreserved in a preset freezer at a temperature of ≤-80°C until collection. The samples are handled in accordance with the separately specified procedures.

Serum Vedolizumab concentration is determined by means of Enzyme-Linked Immunosorbent Assay at an outside laboratory.

9.1.9 α4β7 Receptors Occupancy in Various Lymphocyte Populations in the Colonic Tissue and blood

Lymphocytes are isolated from colon tissue biopsied during colonoscopy using the method of Bowcutt R et al[8)]. Lymphocytes are incubated with multiple antibodies, and after fixation of the cells, they are analyzed by flow cytometry. In addition, 20 mL of venous blood is collected, incubated with antibodies, and analyzed by flow cytometry. These analyses will be performed by an outside laboratory. Detailed procedures will be provided separately.

9.1.10 The Patient Diary

The patient diaries are used for calculation of Mayo scores. At each visit, patient diaries to be entered from the visit to the next visit are handed to subjects. A subject is instructed to record the following items in the diary and bring it at the next visit.

- Stool frequency.
- Rectal Bleeding.
- Use of antidiarrheal drugs.

Contents of the patient diaries are confirmed at each visit. The subject is asked about omissions or inconsistencies for correction, if any. The patient diaries are collected after completion of final confirmation of the contents to be retained.

9.1.11 Complete Mayo Score and Partial Mayo Score

The complete Mayo scores are calculated as total scores of the following 4 subscores in the Table 9.a. The partial Mayo score is calculated as total scores of the following 3 subscores except the subscore for the findings on endoscopy.

Table 9.a Mayo Scoring System for the Assessment of UC Activity

Stool Frequency	
	0: Normal number of stools for this patient
	1: 1 to 2 stools more than normal
	2: 3 to 4 stools more than normal
	3: 5 or more stools more than normal
Rectal Bleeding	
	0: No blood seen
	1: Streaks of blood with stool less than half the time
	2: Obvious blood with stool most of the time
	3: Blood alone passes
Findings on Endoscopy	
	0: Normal or inactive disease
	1: Mild disease (erythema, decreased vascular pattern, mild friability)
	2: Moderate disease (marked erythema, lack of vascular pattern, friability, erosions)
	3: Severe disease (spontaneous bleeding, ulceration)
Physician's Global Assessment	
	0: Normal
	1: Mild disease
	2: Moderate disease
	3: Severe disease

At baseline (VISIT 2), the investigator interviewed about the average number of stools, condition of rectal bleeding, and general condition during the previous 3 days, and evaluated each subscore based on the outcome of the interview to calculate the complete Mayo score.

Two weeks after the first dose of the study drug (VISIT 3), data in the patient diary, the subscores for the findings on endoscopy and physician's global assessment are entered on the (e)CRF to calculate the complete Mayo score or the partial Mayo score. The methodology for calculating each subscore is shown below.

Calculation of Subscores for Stool Frequency and Rectal Bleeding

For calculation of subscores for stool frequency and rectal bleeding, the contents in the patient diary during the previous 7 days before evaluation are entered on the (e)CRF. Each subscore is calculated automatically based on the contents in the patient diary during the last 3 days before evaluation except for the following.

- The day of pretreatment for endoscopy.

- The day of endoscopy and the next day.
- The day of usage of antidiarrheal drugs and the next day.

Calculation of Subscore for Findings on Endoscopy

Endoscopy is performed in the rectum and sigmoid colon between 9 days before and on the day of evaluation of the complete Mayo score. The investigator identifies the most active site at each of Weeks 0 (VISIT 2), 22 (VISIT 6) and 54 (VISIT 10), and the complete Mayo score is calculated from the sum of the subscores of the four items shown in Table 9.a. The partial Mayo score is calculated from the sum of the subscores of three items from these four items, excluding mucosal findings.

Investigator will score the identical site for the findings on endoscopy throughout the study period based on Table 9.a. The findings on endoscopy are scored by the same physician throughout the study, where possible. The obtained subscore for findings on endoscopy is entered on the (e)CRF.

Calculation of Physician's Global Assessment

On the evaluation day, the investigator interviews about abdominal discomfort, general condition, principal physician’s comments, subject’s impression or the like to decide the physician's global assessment as referring the result of the interview, stool frequency, rectal bleeding, and findings on endoscopy (if performed).

9.1.12 AVA/neutralizing AVA

AVA/neutralizing AVA test, 5 mL of the venous blood is collected. At the visit for study drug administration, the blood is to be sampled immediately before administration, where possible. The date and time of the blood sampling are recorded in the (e)CRF.

The collected blood is left standing at room temperature for approximately 30 minutes and centrifuged at 3,000 rpm at 4°C for 10 minutes. The obtained serum is cryopreserved in a preset freezer at a temperature of ≤-80°C until collection. The samples are handled in accordance with the separately specified procedures.

Serum concentration of AVA is determined by means of electrochemoluminescent (ECL) assay at an outside laboratory.

Furthermore, the serum neutralizing AVA is determined only for the AVA-positive samples. The serum neutralizing AVA is also determined by means of ECL assay at an outside laboratory.

9.1.13 Transcriptome Analysis of the Colonic Tissue

Colonic tissue collected during colonoscopy is cryopreserved immediately. Total RNA is isolated from the tissue using the RNeasy Mini Kit. Then total RNA (150 ng) is analyzed for whole-genome gene expression analysis via RNAseq. The detailed procedure for transcriptome analysis is specified in a separate manual.

9.1.14 Immuno-phenotyping in the Colonic Tissue and blood

Lymphocytes are isolated from colon tissue biopsied during colonoscopy using the method of Bowcutt R et al[8)]. Lymphocytes are incubated with multiple antibodies, and after fixation of the cells, they are analyzed by flow cytometry. In addition, 20 mL of venous blood is collected, incubated with antibodies, and analyzed by flow cytometry. These analyses will be performed by an outside laboratory. Detailed procedures will be provided separately.

9.1.15 Biomarkers

All samples are collected in accordance with acceptable laboratory procedures. Biomarkers are listed in Table 9.b.

Table 9.b Biomarkers

**Chemistry**
sMAdCAM-1
CRP
Fecal calprotectin

The central laboratory performs biomarker tests.

9.1.16 Record of Subjects Who Are Withdrawn Before Start of Administration

If the subject is withdrawn before the start of administration of the study drug, the investigator should complete the (e)CRF.

The primary reason for withdrawal before the start of administration is recorded on the (e)CRF using the following categories:

- PTE
- Did not meet inclusion criteria or did meet exclusion criteria
- Significant protocol deviation
- Lost to follow-up

- Voluntary withdrawal <specify reason>
- Study termination
- Others <specify the reason>

The subject identification numbers assigned to subjects withdrawn from the study before the start of administration should not be reused.

9.2 Monitoring Subject Treatment Compliance

The study drug administered to the subjects is recorded in the (e)CRF for the dates, times of start/end of the infusion. When the infusion of the study drug is not completed, dosage and reasons for incomplete dose is recorded in the (e)CRF.

9.3 Schedule of Observations and Procedures

The schedule for all study-related procedures for all evaluations is shown in Appendix A. Assessments should be completed at the designated visit/time points.

9.3.1 At Screening [VISIT 1]

After acquisition of informed consent, a physical examination and tests for screening are performed. Eligibility of subjects is determined in accordance with the inclusion and exclusion criteria as described in Section 7.1 and 7.2, respectively.

Tests and observations to be performed and endpoints to be assessed at screening (VISIT 1) are shown below.

- Informed consent
- Demographics and medication history
- Weight, height and BMI
- Concurrent medical conditions
- Pretreatment events

9.3.2 Baseline [VISIT 2]

Eligibility of subjects is determined in accordance with the inclusion and exclusion criteria as described in Section 7.1 and 7.2, respectively. Tests and observations to be performed and endpoints to be assessed at baseline (VISIT 2) are shown below.

- Physical examination
- Concomitant medications

- Complete Mayo score
- AVA/neutralizing AVA
- Transcriptome analysis of the colonic tissue
- Biomarkers
- Pretreatment events/AEs
- Treatment Compliance

9.3.3 At Week 2 [VISIT 3] and Week 6 [VISIT 4]

Tests and observations to be performed and endpoints to be assessed at Week 2 [VISIT 3] and Week 6 [VISIT 4] are shown below.

- Physical examination
- Concomitant medications
- Check of the patient diary
- Partial Mayo score
- AEs
- Treatment Compliance

9.3.4 At Week 14 [VISIT 5]

Tests and observations to be performed and endpoints to be assessed at Week 14 [VISIT 5] are shown below.

- Physical examination
- Concomitant medications
- Tissue vedolizumab concentration
- Serum vedolizumab concentration
- $\alpha 4\beta 7$ receptors occupancy in various lymphocyte populations in the colonic tissue and blood
- Check of the patient diary
- Complete Mayo score
- AVA/neutralizing AVA

- Transcriptome analysis of the colonic tissue
- Immune-phenotyping
- Biomarkers
- AEs
- Treatment Compliance

9.3.5 At Week 22 [VISIT 6] to 46 [VISIT 9]

Tests and observations to be performed and endpoints to be assessed at Week 22 [VISIT 6] to 46 [VISIT 9] are shown below.

- Physical examination
- Concomitant medications
- Check of the patient diary
- Partial Mayo score
- AEs
- Treatment Compliance

9.3.6 At the End of Study [VISIT 10] or at the Early Termination

A visit in 54 weeks after start of administration is defined as the last VISIT (VISIT 10). Tests and observations to be performed and endpoints to be assessed at the End of Study (VISIT 10) are shown below. At the Early Termination, following procedures will be performed as well.

- Physical examination
- Concomitant medications
- Tissue vedolizumab concentration (Can be omitted without conducting endoscopy in subjects who terminated early)
- Serum vedolizumab concentration
- $\alpha 4\beta 7$ receptors occupancy in various lymphocyte populations in the colonic tissue and blood (Can be omitted without conducting endoscopy in subjects who terminated early)
- Check of the patient diary

- Complete Mayo score (Partial Mayo score is acceptable without conducting endoscopy in subjects who terminated early)
- AVA/neutralizing AVA
- Transcriptome analysis of the colonic tissue (Can be omitted without conducting endoscopy in subjects who terminated early)
- Immune-phenotyping in the colonic tissue and blood (Can be omitted without conducting endoscopy in subjects who terminated early)
- Biomarkers
- AEs

At the end of the study, the status all subjects administered the study drug will be recorded on the (e)CRF.

10.0 ADVERSE EVENTS

10.1 Definitions

10.1.1 PTEs

A PTE is defined as any untoward medical occurrence in a clinical investigation subject who has signed informed consent to participate in a study but prior to the administration of any study drug; it does not necessarily have to have a causal relationship with the study participation.

10.1.2 AEs

An AE is defined as any untoward medical occurrence in a subject administered a pharmaceutical product (including the study drug). It does not necessarily have to have a causal relationship with this pharmaceutical product (including the study drug).

An AE can therefore be any unfavorable and unintended sign (e.g., a clinically significant abnormal laboratory value), symptom, or disease temporally associated with the use of a pharmaceutical product (including the study drug) whether or not it is considered related to the pharmaceutical product (including the study drug).

10.1.3 Additional Points to Consider for PTEs and AEs

An untoward finding generally may:

- Indicate a new diagnosis or unexpected worsening of a pre-existing condition. (Intermittent events for pre-existing conditions or underlying disease should not be considered PTEs or AEs.).
- Necessitate therapeutic intervention.
- Require an invasive diagnostic procedure.
- Require discontinuation or a change in the dose of the study drug, or concomitant medication.
- Be considered unfavorable by the investigator for any reason.

PTEs/AEs caused by a study procedure (e.g., a bruise after blood draw) should be recorded as a PTE/AE.

Diagnosis vs signs and symptoms:

- Each event should be recorded to represent a single diagnosis. Accompanying signs (including abnormal laboratory values or ECG findings) or symptoms should NOT

be recorded as additional AEs. If a diagnosis is unknown, sign(s) or symptom(s) should be recorded appropriately as a PTE(s) or as an AE(s).

Laboratory values and ECG findings:

- Changes in laboratory values or ECG findings are only considered to be PTEs or AEs if they are judged to be clinically significant (i.e., if some action or intervention is required or if the investigator judges the change to be beyond the range of normal physiologic fluctuation). A laboratory or ECG re-test and/or continued monitoring of an abnormal value or findings are not considered an intervention. In addition, repeated or additional noninvasive testing for verification, evaluation or monitoring of an abnormality is not considered an intervention.
- If abnormal laboratory values or ECG findings are the result of pathology for which there is an overall diagnosis (e.g., increased creatinine in renal failure), the diagnosis only should be reported appropriately as a PTE or as an AE.

Pre-existing conditions (a disease or symptom that is present at the start of the study drug administration):

- Pre-existing conditions are considered concurrent medical conditions and should NOT be recorded as PTEs or AEs. Baseline evaluations (e.g., laboratory tests, ECG, X-rays etc.) should NOT be recorded as PTEs unless related to study procedures. However, if the subject experiences a worsening or complication of such a concurrent medical condition, the worsening or complication should be recorded appropriately as a PTE (worsening or complication occurs before start of study drug) or as an AE (worsening or complication occurs after start of study drug). Investigators should ensure that the event term recorded captures the change in the condition (e.g., “worsening of…”).
- If a subject has a pre-existing episodic concurrent medical condition (e.g., asthma, epilepsy) any occurrence of an episode should only be captured as a PTE/AE if the condition becomes more frequent, serious or severe in nature. Investigators should ensure that the AE term recorded captures the change in the condition from baseline (e.g., “worsening of…”).
- If a subject has a degenerative concurrent medical condition (e.g., cataracts, rheumatoid arthritis), worsening of the condition should only be recorded as a PTE/AE if occurring to a greater extent to that which would be expected. Investigators should ensure that the AE term recorded captures the change in the condition (e.g., “worsening of…”).

Worsening of PTEs or AEs:

- If the subject experiences a worsening or complication of a PTE after the start of study drug, the worsening or complication should be recorded as an AE. Investigators should ensure that the AE term recorded captures the change in the PTE (e.g., "worsening of…").
- If the subject experiences a worsening or complication of an AE after any change in study drug, the worsening or complication should be recorded as a new AE. Investigators should ensure that the AE term recorded captures the change in the condition (e.g., "worsening of…").

Changes in intensity of AEs /serious PTEs:

- If the subject experiences changes in intensity of an AE/serious PTE, the event should be captured once with the maximum intensity recorded.

Preplanned procedures (surgery or interventions):

- Preplanned procedures (surgeries or therapies) that are scheduled prior to signing of informed consent are not considered PTEs or AEs. However, if a preplanned procedure is performed early (e.g., as an emergency) due to a worsening of the pre-existing condition, the worsening of the condition should be recorded as a PTE or an AE. Complications resulting from any planned surgery should be reported as AEs.

Elective surgeries or procedures:

- Elective procedures performed where there is no change in the subject's medical condition should not be recorded as PTEs or AEs but should be documented in the subject's source documents. Complications resulting from an elective surgery should be reported as AEs.

Insufficient clinical response (lack of efficacy):

- Insufficient clinical response, efficacy, or pharmacologic action should NOT be recorded as an AE. The investigator must make the distinction between exacerbation of pre-existing illness and lack of therapeutic efficacy.

Overdose:

- Cases of overdose with any medication without manifested side effects are NOT considered AEs, but instead will be documented on an Overdose page of the

(e)CRF. Any manifested side effects will be considered AEs and will be recorded on the AE page of the (e)CRF.

10.1.4 SAEs

An SAE is defined as any untoward medical occurrence that at any dose:

1. Results in death,
2. Is life threatening*,
3. Requires inpatient hospitalization or prolongation of existing hospitalization,
4. Results in persistent or significant disability/incapacity,
5. Leads to a congenital anomaly/birth defect,
6. Is an important medical event that may expose the subject to danger even though the event is not immediately life-threatening or fatal does not result in hospitalization, or requires intervention to prevent items 1 through 5 above.

* The term “life threatening” refers to an event in which the subject is at risk of death at the time of the event; it does not refer to an event that hypothetically might have caused death if it is more severe.

10.1.5 AEs of particular interest

Adverse events of particular interest (whether serious or not) are those events of scientific and medical concern that are specific to the study drug or clinical research in question. In the event of Infusion reaction including hypersensitivity reactions, infections, progressive multifocal leukoencephalopathy, and malignancies, the investigator or subinvestigator will provide detailed information upon request from Takeda.

10.1.6 Intensity of PTEs and AEs

The different categories of intensity (severity) are characterized as follows.

Mild	The event is transient and easily tolerated by the subject.
Moderate	The event interrupts the subject’s usual activities.
Severe	The event causes considerable interference with the subject’s usual activities.

10.1.7 Causality of AEs

The relationship of each AE to the study drug will be assessed using the following categories.

Related	An AE that follows a temporal sequence (including clinical course after discontinuation), or an AE in which there is at least a reasonable probability that a causal relationship to the study drug cannot be ruled out, although other factors such as underlying disease, complications, or concomitant drugs/treatment are also suspected.
Not related	An AE that does not follow a temporal sequence from administration of the study drug or comparative drug. Very likely due to other factors such as underlying disease, complications, or concomitant drugs/treatment.

10.1.8 Relationship to Study Procedures

Relationship (causality) to study procedures should be determined for all PTEs and AEs.

The relationship should be assessed as Related if the investigator considers that there is reasonable possibility that an event is due to a study procedure. Otherwise, the relationship should be assessed as Not Related.

10.1.9 Start Date

The start date of the AE/PTE is the date that the first signs/symptoms are noted by the subject and/or investigator.

The start date of PTEs/AEs will be determined using the following criteria.

PTEs/AEs	Start date
Signs, symptoms, diseases (diagnoses)	The date on which the first signs/symptoms are noted by the subject and/or the investigator.
Asymptomatic diseases	The date on which a diagnosis is confirmed through a test(s).<br>The date on which a diagnosis is confirmed, even when the test results indicate an old sign(s) of the disease or an approximate time of its onset.
Exacerbation of comorbidities	The date on which the first worsening of diseases/symptoms is noted by the subject and/or the investigator.
Onset of a test abnormality after the start of study drug administration	The date on which a clinically significant laboratory abnormality is detected.
Worsening of a baseline test abnormality after the start of study drug administration	The date on which a clear increase/decrease in a laboratory parameter is clinically confirmed based on the time profile of the parameter.

10.1.10 Stop Date

The stop date of the PTE/AE is the date at which the subject recovered, the event resolved but with sequelae or the subject died.

10.1.11 Frequency

Episodic AEs/PTE (e.g., vomiting) or those which occur repeatedly over a period of consecutive days are intermittent. All other events are continuous.

10.1.12 Action Concerning Study Drug

Action concerning study drug will be classified or defined as shown below.

Drug withdrawn	The study drug is discontinued because of an AE (including withdrawal by the subject at his/her discretion).<br>If the study drug is continued after the study termination, the action should be "Dose not changed".
Dose not changed	The dose is not changed after the onset of the AE.<br>The study drug is discontinued, reduced, or increased because of another AE.<br>The study drug is discontinued or reduced for a reason other than the AE, e.g., inadvertence of the subject.
Unknown	It has not been possible to determine what action has been taken because the subject is lost to follow-up.
Not Applicable	The administration of the study drug had already been completed or discontinued before the onset of the AE.
Dose reduced	The dose of the study drug is reduced because of the AE.
Drug increased	The dose of the study drug is increased because of the AE..
Washout	The study drug is suspended (i.e., interrupted) because of the AE (including suspension/interruption by the subject at his/her discretion), but resumed later.

10.1.13 Outcome

Outcome of AEs is classified as follows:

Recovered/ Resolved	Subject returned to first assessment status with respect to the AE/PTE.
Recovering/ Resolving	The intensity is lowered by 1 or more stages: the diagnosis or signs/symptoms has almost disappeared; the abnormal laboratory value improved but has not returned to the normal range or to baseline; the subject died from a cause other than the particular AE/PTE with the condition remaining "recovering/resolving".

Not recovered/ not resolved	There is no change in the diagnosis, signs or symptoms; the intensity of the diagnosis, signs/ symptoms or laboratory value on the last day of the observed study period has got worse than when it started; is an irreversible congenital anomaly; the subject died from another cause with the particular AE/PTE state remaining "Not recovered/not resolved".
Resolved with sequelae	The subject recovered from an acute AE/PTE but is left with permanent/significant impairment (e.g., recovered from a cardiovascular accident but with some persisting paresis.
Fatal	The AEs/PTEs which are considered as the cause of death.
Unknown	The course of the AE/PTE cannot be followed up due to hospital change or residence change at the end of the subject's participation in the study.

10.2 Procedures

10.2.1 Collection of AEs

*10.2.1.1 **PTE and AE Collection Period***

Collection of PTEs will commence from the time the subject signs the informed consent to participate in the study and continue until the subject is first administered study drug (VISIT 2) or until screen failure. For subjects who discontinue prior to study drug administration, PTEs are collected until the subject discontinues study participation.

Collection of AEs will commence from the time that the subject is first administered study drug (VISIT 2). Routine collection of AEs will continue until VISIT 10.

*10.2.1.2 **Collection of PTE and AE***

At each study visit, the investigator will assess whether any subjective AEs have occurred. A neutral question, such as "How have you been feeling since your last visit?". Subjects may report AEs occurring at any other time during the study. Subjects experiencing a PTE related to the study procedure must be monitored until the symptoms subside and any clinically relevant changes in laboratory values have returned to baseline or there is a satisfactory explanation for the change. PTEs unrelated to the study procedure need not to be followed-up for the purposes of the protocol.

All subjects experiencing AEs, whether considered associated with the use of the study drug or not, must be monitored until the symptoms subside and any clinically relevant changes in laboratory values have returned to baseline or until there is a satisfactory explanation for the changes observed. All AEs will be documented in the PTE/AE page of the (e)CRF, whether the investigator concludes that the event is related to the drug treatment. The following information will be documented for each event:

1. Event term.

2. Start and stop date.
3. Frequency
4. Intensity.
5. Investigator's opinion of the causal relationship between the event and administration of the study drug (related or not related) (not completed for PTEs).
6. Investigator's opinion of the causal relationship to study procedures, including the detail of the suspected procedure.
7. Action concerning study drug (not applicable for PTEs).
8. Outcome of event.
9. Seriousness.

Infusion reactions (e.g., rash, pruritus, flushing, or urticaria) which are observed during infusion and 1 hour after infusion of the study drug is recorded in the (e)CRF.

Patient diaries will not be used as the primary method for collecting adverse events. However, if the principal investigator or research associate recognizes a potential adverse event based on information collected using this tool, appropriate follow-up will be conducted on the subject for medical evaluation. If the follow-up results indicate that the event is an adverse event that has not been previously reported, the event will be reported in accordance with normal reporting procedures.

10.2.1.3 AEs of particular interest

Adverse events of particular interest should be recorded in the case report form as adverse events. If requested by Takeda, the principal investigator or a sub-investigator will submit a detailed information report to Takeda.

10.2.2 Reporting of SAEs

When SAE occurs through the AE collection period it should be reported according to the following procedure:

SAE should be reported by the investigator to Takeda immediately along with any relevant information. The investigator should submit the detailed SAE Form to Takeda within 10 calendar days. The information should be completed as fully as possible but contain, at a minimum:

- A short description of the event and the reason for why the event is categorized as serious.
- Subject identification number.
- Investigator's name.
- Name of the study drug.
- Causality assessment.

Any SAE spontaneously reported to the investigator following the AE collection period should be reported to Takeda if considered related to study participation.

10.2.3 Reporting of Non-serious AEs

Non-serious AE should be reported by the investigator to Takeda. The investigator submits information to the (e)CRF within the period specified by Takeda upon request from Takeda.

10.2.4 Reporting of Special Situation

When special situation listed in Appendix B is found, the investigator should report to Takeda immediately.

10.2.5 Abnormal liver function tests

In case of ALT or AST >3 × ULN and total bilirubin >2 × ULN as a result of liver function tests performed as part of routine medical care, and if these abnormal liver function tests cannot be explained by other factors, the event should be recorded as a serious adverse event and reported according to Section 10.2.2. Report it in accordance with Section 10.2.2. The investigator will report to the monitor/Takeda and confirm detailed information on the subject and possible factors other than the study drug (acute viral hepatitis A or B, other acute liver diseases, etc., and history/complications). The criteria are different from those for abnormal liver function test values to consider discontinuation as indicated in Section 7.5.1.

10.2.6 Follow-up of AEs

If information not available at the time of the first report becomes available later, the investigator should complete a follow-up SAE form or provide other written documentation and submit it to Takeda. Copies of any relevant data from the hospital notes (e.g., ECGs, laboratory tests, discharge summary, postmortem results) should be sent to Takeda, if requested.

All AEs should be followed up until resolution or permanent outcome of the event. The timelines and procedure for follow-up reports are the same as those for the initial report.

10.2.7 Reporting of Additional Information on Adverse Events

If Takeda requests additional information regarding an adverse event for reporting to regulatory authorities, the investigator or sub-investigator shall review the necessary additional information and enter it into the electronic case report system or submit a written report within the timeframe specified by Takeda.

10.3 Reporting of Disease or the Like

10.3.1 Reporting of Disease or the Like to CRB

The investigator notifies disease or the like (any disease, disability, death or infection which is suspected of being due to the conduct of the study) to the head of study site and the principal investigator according to procedure described in separate document.

In case of SAE, the principal investigator consults CRB and notify to all investigators of study sites. In case of non-SAE, the principal investigator reports to CRB as a part of periodical report and notifies to all investigators of study sites.

The investigator should report to the head of study site.

10.3.2 Reporting of Safety Data to Regulatory Authority

Takeda reports the suspected, unexpected, serious adverse reaction and other SAE required expedited reporting to the regulatory authority in accordance with regulations.

From the time that Takeda or a CRO commissioned by Takeda first knows the event or obtains additional information, it will comply with the reporting deadline specified in the regulatory requirements and make an urgent report to the regulatory authority.

11.0 STUDY-SPECIFIC COMMITTEES

No committees are set for this study.

12.0 DATA HANDLING AND RECORDKEEPING

The full details of procedures for data handling is documented in the Data Management Plan. AEs, medical history, and concurrent medical conditions is coded using the Medical Dictionary for Regulatory Activities (MedDRA). Drugs are coded using the World Health Organization (WHO) Drug Dictionary.

12.1 CRFs (Electronic)

Completed (e)CRFs are required for each subject who signs an informed consent.

The principal investigator or its designee supplies study sites with access to (e)CRFs. These forms are used to transmit the information collected in the performance of this study to the sponsor and regulatory authorities. (e)CRFs must be completed in Japanese. Data are transcribed directly onto (e)CRFs.

After completion of the entry process, computer logic checks are run to identify items, such as inconsistent dates, missing data, and questionable values.

Corrections are recorded in an audit trail that captures the old information, the new information, identification of the person making the correction, the date the correction is made, and the reason for change.

The investigator must review the (e)CRFs for completeness and accuracy and must e-sign the appropriate (e)CRFs as indicated. Furthermore, the investigator must retain full responsibility for the accuracy and authenticity of all data entered on the (e)CRFs.

The following data will be recorded directly into the (e)CRFs:

- Date, start time and end time of administration of the study drug
- Dose and reason for failure to complete the infusion (if the infusion of the study drug was not completed)
- Severity, degree, the causal relationship with the study drug, or the study procedures, outcome

The following data will not be recorded into the (e)CRFs:

- BMI
- Tissue vedolizumab concentration
- Serum vedolizumab concentration
- Complete Mayo score

- α4β7 Receptors Occupancy in Various Lymphocyte Populations in the Colonic Tissue
- Transcriptome analysis of the colonic tissue
- Biomarkers

After the lock of the study database, any change of, modification of or addition to the data on the (e)CRFs should be made by the investigator with use of change and modification records of the (e)CRFs. The investigator must review the data change for completeness and accuracy, and must sign, or sign and seal, and date.

(e)CRFs are reviewed for completeness and acceptability at the study site during periodic visits by the principal investigator or its designee. The principal investigator or its designee is permitted to review the subject's medical and hospital records pertinent to the study to ensure accuracy of the (e)CRFs. The completed (e)CRFs are the property of the principal investigator and Takeda and should not be made available in any form to third parties, except for authorized representatives of appropriate governmental health or regulatory authorities, without written permission of the principal investigator and Takeda.

12.2 Record Retention

The investigator and the head of the study site agree to keep the records stipulated in Section 12.1 and those documents that include (but are not limited to) the study-specific documents, the identification log of all participating subjects, medical records, all original signed and dated informed consent forms, and electronic copy of eCRFs, including the audit trail to enable evaluations or audits from regulatory authorities, the principal investigator or its designees.

The investigator and the head of the study site are required to retain essential relevant documents until the day 5 years after the date of early termination or completion of the study. However, if the principal investigator or Takeda request a longer time period for retention, the head of the study site should discuss how long and how to retain those documents with the principal investigator or Takeda.

13.0 STATISTICAL METHODS

13.1 Statistical and Analytical Plans

First version of statistical analysis plan (SAP) is prepared before the informed consent of the first subject. This document provides further details regarding the definition of analysis variables and analysis methodology to address all study objectives. SAP is revised and finalized prior to the database lock when needed. The statistical analysis is conducted after the database lock according to SAP.

13.1.1 Analysis Sets

In this study, full analysis set (FAS) is defined. The FAS, the main analysis set used for primary efficacy analysis, is defined as "all subjects who received at least one dose of the study drug for the treatment period." The definition of analysis set is described in the Handling Rules for Analysis Data.

The person in charge of analysis verifies the validity of the definitions of the analysis sets as well as the rules for handling data, with consulting a medical expert as needed. If necessary, the Handling Rules for Analysis Data is supplemented with new handling rules that are not discussed at the planning stage. The Handling Rules for Analysis Data must be finalized prior to database lock.

13.1.2 Analysis of Demographics and Other Baseline Characteristics

Demographics and other baseline characteristics are summarized in FAS.

13.1.3 Pharmacokinetics and Efficacy Analysis

(1) Primary Endpoint and Analysis Methodology

[Primary endpoint]

- Correlation between the concentrations of vedolizumab in the colonic tissue and in the serum, and clinical remission at Week 54

[Analysis methodology]

Following analyses are conducted using FAS.

Calculate the remission rate when stratified in 4 categories with the first quartile, median, and third quartile of vedolizumab concentration in colon tissue (inflamed / non-inflamed) at week 54 as the boundaries. Calculate summary statistics for vedolizumab concentrations in colon tissue and serum by remission at week 54.

(2) Secondary Endpoints and Analysis Methodology

[Secondary endpoints]

- Concentrations of vedolizumab in the colonic tissue and in the serum, and clinical remission rate at Week 14
- Correlation between the concentrations of vedolizumab in the colonic tissue and in the serum, and mucosal healing at Week 14 and 54
- Correlation between the α4β7 integrin receptor occupancy and clinical outcomes (clinical remission and mucosal healing) at Week 14 and 54
- Correlation between the colonic tissue and serum concentrations of vedolizumab and α4β7 integrin receptor occupancy at Week 14 and 54
- Concentrations of vedolizumab in the colonic tissue and in the serum at Week 14 and 54
- α4β7 receptors occupancy in various lymphocyte populations in the colonic tissue and in the blood at Week 14 and 54
- Proportion of subjects achieving clinical remission and mucosal healing at Week 14 and 54
- Correlation between the concentrations of vedolizumab in the colonic tissue and in the serum, α4β7 integrin receptor occupancy and clinical outcomes in subjects with or without previous exposure to TNFα antagonist at Week 14 and 54
- Concentrations of vedolizumab in the colonic tissue and in the serum in subjects with or without previous exposure to TNFα antagonist at Week 14 and 54
- Proportion of subjects achieving clinical remission and mucosal healing in subjects with or without previous exposure to TNFα antagonist at Week 14 and 54
- Correlation between AVA and neutralizing AVA and clinical outcomes at Week 14 and 54
- Proportion of subjects positive for AVA and neutralizing AVA at Baseline, Week

[Analysis methodology]

Following analysis are conducted using FAS.

- Concentrations of vedolizumab in the colonic tissue and in the serum and clinical remission at Week 14 will be tabulated in a manner consistent with that used for the primary endpoint.

- Concentrations of vedolizumab in the colonic tissue and in the serum and mucosal healing at Week 14 and 54 will be tabulated in a manner consistent with that used for the primary endpoint.
- The α4β7 integrin receptor occupancy and clinical outcomes at Week 14 and 54 will be tabulated in a manner consistent with that used for the primary endpoint.
- Correlation analysis between concentrations of vedolizumab in the colonic tissue and in the blood and α4β7 receptors occupancy at Week 14 and 54 are performed.
- Summary statistics will be calculated for concentrations of vedolizumab in the colonic tissue and in the serum at Week 14 and 54, and time profiles (individual and mean/standard deviation) are plotted.
- Correlation analysis between colon tissue and serum vedolizumab concentrations are performed at Weeks 14 and 54.
- Summary statistics will be calculated for α4β7 integrin receptor occupancy in various lymphocytes in colon tissue and blood at Weeks 14 and 54, and time profiles (individual and mean/standard deviation) are plotted.
- The rates of clinical remission and mucosal healing at Weeks 14 and 54.
- For the complete Mayo score, partial Mayo score, and Mayo endoscopic subscore, summary statistics are calculated at baseline, Week 14, and Week 54. In addition, summary statistics of the change from baseline to Week 14 and Week 54 are calculated, and mean plots with SD will be prepared.
- Subgroup analyses are performed in TNF-α antagonist-naïve subjects and TNF-α antagonist-failure subjects in terms of correlation between vedolizumab concentrations in colon tissue and serum, α4β7 receptor saturation, and clinical outcomes at Weeks 14 and 54 and their relationship.
- Clinical remission and mucosal healing are calculated by AVA/neutralizing AVA or not at Week 14 and 54.
- Proportions of subjects positive for AVA and neutralizing AVA/neutralizing AVA are summarized at Week 14 and 54.

(3) Exploratory Endpoints

See Section 5.2.3.

(4) Data Transformation and Missing Data

Details are specified in the “Statistical Analysis Plan.”

In the analysis of the efficacy endpoints of clinical remission and mucosal healing, when adjudications for these endpoints are missing at the time of evaluation, not-remission or not-healing are imputed. If the vedolizumab concentration in colon tissue or serum or α4β7 receptor occupancy is missing, it is excluded from the analysis at that time point.

(5) Significance Level and Confidence Coefficient

Confidence interval (CI): 95% (two-sided estimation)

13.1.4 Safety Analysis

AEs are summarized using the FAS.

All AEs are coded using MedDRA. Data are summarized using preferred term and primary system organ class.

- Incidence of all AEs
- Incidence of AEs related to the study drug
- Incidence of all AEs by severity
- Incidence of AEs related to the study drug by severity
- Incidence of AEs leading to discontinuation of study treatment
- Incidence of serious AEs

13.2 Interim Analysis and Criteria for Early Termination

No interim analysis is planned.

13.3 Determination of Sample Size

30 subjects

<Rationale for the number of planned subjects>

A sample size of 30 subjects was defined based on feasibility reasons.

14.0 QUALITY CONTROL AND QUALITY ASSURANCE

14.1 Study-Site Monitoring Visits

Monitoring visits to the study site are periodically performed during the study to ensure that all aspects of the protocol are followed. Source documents are reviewed for verification of data recorded on the (e)CRFs. Source documents are defined as original documents, data, and records. The investigator and the study site guarantee access to source documents by the principal investigator, Takeda or its designee (CRO).

All aspects of the study and its documentation are subject to be reviewed by the principal investigator, Takeda or its designee, including but not limited to the identification log of all participating subjects, subject medical records, informed consent documentation, and review of (e)CRFs and associated source documents. It is important that the investigator and other study personnel are available during the monitoring visits and that enough time is devoted to the process.

14.2 Protocol Deviation

The investigator can deviate from the protocol for any medically unavoidable reason, for example, to eliminate an immediate hazard to study subjects, without a prior written agreement with the principal investigator and Takeda or a prior approval from CRB. In the event of a deviation or change, the investigator should notify the principal investigator and the head of the study site of the deviation or change as well as its reason in a written form, and then retain a copy of the written form. When necessary, the investigator may consult and agree with the principal investigator and Takeda on a protocol amendment. If the protocol amendment is appropriate, the amendment proposal should be submitted to the head of the study site as soon as possible and an approval from CRB should be obtained.

The investigator should document all protocol deviations.

If the sub-investigator becomes aware of the occurrence of a condition not conforming to the Clinical Research Act or this protocol (non-conformance), the subinvestigator will promptly report it to the investigator. If the investigator becomes aware of the occurrence of non-conformance, he/she will promptly report it to the manager of the study site and notify it to the principal investigator. The principal investigator shall promptly provide information on the occurrence of non-conformance to other investigators, and if any particularly significant non-conformance is found, he/she shall promptly seek the opinion of the CRB.

14.3 Quality Assurance Audits and Regulatory Agency Inspections

The study site also may be subject to quality assurance audits by the principal investigator, Takeda or designees. In this circumstance, the designated auditor contacts the site in advance to arrange an auditing visit. The auditor may ask to visit the facilities where laboratory samples are collected, where the medication is stored and prepared, and any other facility used during the study. In addition, there is the possibility that this study may be inspected by CRB or regulatory agencies. If the study site is contacted for an inspection by a regulatory body, the principal investigator and Takeda should be notified immediately. The investigator and head of the study site guarantee access for quality assurance auditors to all study documents as described in Section 14.1.

15.0 ETHICAL ASPECT OF THE STUDY

This study is conducted with the highest respect for the individual participants (i.e., subjects) according to the protocol, the ethical principles that have their origin in the Declaration of Helsinki, and the Clinical Trials Act. Each investigator conducts the study according to applicable local regulatory requirements and align his or her conduct in accordance with the “Responsibilities of the Investigator” that are listed in Appendix C. The principles of Helsinki are addressed through the protocol and through appendices containing requirements for informed consent and investigator responsibilities.

15.1 CRB Approval

The principal investigator or designee supplies relevant documents for submission to CRB for review and approval of the study plan. The study plan (including the informed consent form), this protocol, summary of the study drug and other documents required by all applicable laws and regulations, must be submitted to CRB.

The principal investigator or the designee submit the study plan approved by CRB to MHLW before commencement of the study.

The investigator does not initiate any study procedure such as subject selection prior the approval of the study implementation by the head of the study site and prior the notification from the principal investigator that the study plan was submitted to the MHLW (or confirmation that disclosure in the Japan Registry of Clinical Trials [jRCT]).

The investigator must adhere to all requirements stipulated by CRB. This may include notification to CRB regarding protocol amendments, safety reporting requirements, reports and updates regarding the ongoing review of the study. All CRB approvals and relevant documentation for these items must be provided to the principal investigator or its designee. Payments for Burden Reduction to the subjects shall be limited to a level that does not create an undue expectation of participation. Payment of the Burden Reduction Payment to research subjects must be approved by an accredited clinical research review committee and Takeda.

15.2 Subject Information, and Informed Consent

Written consent documents embody the elements of informed consent as described in the Declaration of Helsinki and the Clinical Trials Act and are in accordance with all applicable laws and regulations. The informed consent form describes the planned and

permitted uses, transfers, and disclosures of the subject's personal and personal health information for purposes of conducting the study. The informed consent form further explains the nature of the study, its objectives, and potential risks and benefits, as well as the date informed consent is given. The informed consent form details the requirements of the participant and the fact that he or she is free to withdraw at any time without giving a reason and without prejudice to his or her further medical care.

The principal investigator is responsible for the preparation, content, and CRB approval of the informed consent form. The informed consent form must be approved by Takeda prior to its submission to the CRB.

The informed consent form must be written in a language fully comprehensible to the prospective subject. It is the responsibility of the investigator to explain the detailed elements of the informed consent form to the subject. Information should be given in both oral and written form whenever possible and in the manner deemed appropriate by CRB.

The subject and the subject's legally acceptable representative must be given ample opportunity to: (1) inquire about details of the study and (2) decide whether to participate in the study. If the subject and the subject's legally acceptable representative determines he or she will participate in the study, then the informed consent form must be signed and dated by the subject and the subject's legally acceptable representative at the time of consent and prior to the subject entering into the study. The subject should be instructed to sign using their legal names, not nicknames, using blue or black ballpoint ink. The investigator must also sign and date the informed consent form at the time of consent and prior to subject entering into the study.

Once signed, the original informed consent form is stored in the investigator's site file. The investigator must document the date the subject signs the informed consent in the subject's medical record. Copies of the signed informed consent form shall be given to the subject.

In case the informed consent form used at each study site is changed from the CRB approved informed consent form, the investigator submits the revised informed consent form to the principal investigator. The principal investigator consults to CRB and/or the Regional Health and Welfare Bureau and takes appropriate procedure (such as CRB approval and submit revised Study Plan to MHLW). The principal investigator or sub-investigator may not use the revised consent and explanation document until he/she is notified by the principal investigator of the completion of the revision procedure.

15.3 Procedures for Study Plan Revision

When it becomes necessary to revise a document that has been approved by CRB, the principal investigator and Takeda consider and decide whether to revise the document.

Approval process of CRB committee regarding the revised document is the same as in Section 15.1.

When the protocol is revised, all study sites conduct research based on the revised protocol after the enforcement date.

All revised informed consent forms must be reviewed and signed by relevant subjects in the same manner as the original informed consent. The date the revised consent is obtained should be recorded in the subject's medical record, and the subject should receive a copy of the revised informed consent form.

15.4 Subject Confidentiality

The principal investigator, Takeda and designees affirm and uphold the principle of the subject's right to protection against invasion of privacy. Throughout this study, a subject's source data only links to the clinical study database or documentation via a subject identification number. As permitted by all applicable laws and regulations, limited subject attributes, such as sex, age, or date of birth may be used to verify the subject and accuracy of the subject's unique identification number.

To comply with the Clinical Trials Act and to verify compliance with this protocol, the principal investigator and Takeda requires the investigator to permit the monitor or the designee, representatives from any regulatory authority, the auditors designated by the principal investigator or Takeda, and CRB to review the subject's original medical records (source data or documents), including, but not limited to, laboratory test result reports, ECG reports, admission and discharge summaries for hospital admissions occurring during a subject's study participation, and autopsy reports. Access to a subject's original medical records requires the specific authorization of the subject as part of the informed consent process (see Section 15.2).

Copies of any subject source documents that are provided to the principal investigator and Takeda must have certain personally identifiable information removed (i.e., subject name, address, and other identifier fields not collected on the subject's [e]CRF).

15.5 Conflict of Interests

This study is conducted with the support of Takeda.

The investigator creates and submits to the principal investigator the conflict of interest management plan that sets out the appropriate handling of donations, honoraria for writings, lectures and other involvement from Takeda for the person who is engaged in the study (the investigator or the person responsible for statistical analysis) and the person described in this protocol those who are apparently profitable by implementing this study.

The principal investigator obtains the opinion of CRB regarding the conflict of interest management standards and the conflict of interest management plan prepared by each study site.

The principal investigator will appropriately manage conflicts of interest based on the conflict of interest management standards and the conflict of interest management plan approved by CRB.

15.6 Financial Burden

Of the expenses for this study, Takeda pays for medical treatment not covered by health insurance. The subjects pay expenses for medical treatment covered by ordinary health insurance.

The subjects are paid a compensation according to the provisions of informed consent form.

15.7 Benefits and Inconveniences to Subjects

15.7.1 Benefits to Subjects

The subjects obtain detailed information on the status of their UC through participation in this study.

15.7.2 Inconveniences to Subjects

Participation in this study may generatein assumed as follows.

・Possibility of temporal burden (approximately 5 minutes) and bleeding from the biopsy site due to longer examination time compared to usual medical care to biopsy the colon tissue during colonoscopy.

・An increase in the blood sampling volume (approximately 50 mL per sampling) by performing blood sampling at Weeks 0, 14, and 54 (or at discontinuation) for the measurement of serum vedolizumab concentrations, AVA, α4β7 receptor occupancy, immunophenotypes of various lymphocytes, and biomarkers.

・Time burden of completing the patient diary every day from 1 week before the next scheduled visit to the visit (about 10 minutes a day).

15.7.3 Ethical Considerations for Study Design

Study drug is a marketed product, and serious disadvantages are not expected from the study. If any health injury occurs during or after the clinical research, the physician will provide appropriate medical examination and treatment within the scope of medical insurance. In addition, in association with the implementation of this study, clinical study liability insurance will be purchased.

Information on benefits, burdens, and expected disadvantages that will be caused to subjects by participation in this study will be provided to candidate subjects, and only subjects who fully understand and agree to these will be enrolled. During the study, all information that may affect subjects' willingness to continue participation in the study will be provided to the subjects.

The investigator informs the patient that participation is voluntary and that the patient may withdraw consent at any time with no reason given and without penalty or loss of benefits to which the patient otherwise is entitled.

The samples and information obtained in this study will be strictly protected and controlled in accordance with the standards ("Act on the Protection of Personal Information "," Clinical Research Act ", etc.) specified by the government.

15.8 Attribution of Study Results

The study results and data obtained from this study belong to the representative institution. Takeda retains the right to use data for free.

The data obtained in this study may be used for secondary use (meta-analysis, etc.) under condition that the data not be linked to personal identification information.

15.9 Publication, Disclosure, and Clinical Trial Registration Policy

15.9.1 Publication and Disclosure

The investigator is obliged to provide the principal investigator and Takeda with complete test results and all data derived by the investigator from the study. During and after the study, only the principal investigator and Takeda may make study information available to other study investigators or to regulatory agencies, except as required by law or regulation. Except as otherwise allowable in the study site agreement, any public

disclosure (excluding disclosure in jRCT) related to the protocol or study results is the sole responsibility of Takeda.

Takeda may publish any data and information from the study (including data and information generated by the investigator) without the consent of the investigator. Authorship of manuscript and congress presentation will be determined according to The International Committee of Medical Journal Editors recommendation for authorship criteria. All publications and presentations must be prepared in accordance with this section and the study site agreement. In the event of any discrepancy between the protocol and the study site agreement, the study site agreement will prevail.

Takeda may publish any data and information from the study (including data and information generated by the investigator) without the consent of the investigator.

The investigator needs to obtain a prior written approval from Takeda to publish any information from the study externally such as to a professional association.

15.9.2 Clinical Study Registration

The principal investigator registers and discloses information relating this study in jRCT. In order to ensure that information on clinical trials reaches the public in a timely manner and to comply with applicable laws, regulations and guidance, Takeda registers all interventional clinical trials it sponsors anywhere in the world on ClinicalTrials.gov before start of study, as defined in Takeda Policy/Standard. Takeda contact information, along with investigator's city, country, and recruiting status is registered and available for public viewing.

15.9.3 Clinical Study Results Disclosure

In addition to clinical study results disclosure to jRCT by the principal investigator, Takeda will post the results of clinical trials on ClinicalTrials.gov, as required by Takeda Policy/Standard, applicable laws and/or regulations.

15.10 Insurance and Compensation for Injury

Takeda or its designee will obtain clinical study insurance against the risk of injury to study subjects. If an adverse event occurs during this study, the investigator or sub-investigator will promptly take necessary actions (Test, treatment, discontinuation of clinical research, etc.) to ensure the safety of subjects. Even if there is no legal responsibility, the following compensation will be provided by the clinical research

liability insurance. The compensation principle shall not preclude subjects from exercising their right to claim for damages.

・Compensation when disability of Grade 1 to 3 or death occurs due to clinical research (disability compensation or bereaved family compensation)

・Medical expenses and medical allowance when hospitalization or treatment equivalent to hospitalization is required due to adverse reactions (unknown or known)

Reimbursement will be provided to reduce the subject's burden (Section 15.7.2) other than insurance. If the investigator has questions regarding this policy, he or she should contact Takeda or its designee.

16.0 REFERENCES

1. Diagnostic Criteria and Treatment Guideline for Ulcerative Colitis and Crohn's Disease. Research Group for Intractable Inflammatory Bowel Disease Designated by the MHLW of Japan (the Group of Dr. Watanabe), the additional volume of Assigned Study Report in 2012.

2. Soler D, Chapman T, Yang LL, et al. The binding specificity and selective antagonism of vedolizumab, an anti-alpha4beta7 integrin therapeutic antibody in development for inflammatory bowel diseases. J Pharmacol Exp Ther. 2009; 330:864-75.

3. Feagan BG, Rutgeerts P, Sands BE, et al. Vedolizumab as induction and maintenance therapy for ulcerative colitis. N Engl J Med. 2013; 369:699-710.

4. Motoya S, Watanabe K, Ogata H, et al. Vedolizumab in Japanese patients with ulcerative colitis: A Phase 3, randomized, double-blind, placebo-controlled study. PLoS One. 2019; 14:e0215491. doi: 10.1371/journal.pone.0215491.

5. Sands BE, Peyrin-Biroulet L, Loftus EV Jr, et al. Vedolizumab versus adalimumab for moderate-to-severe ulcerative colitis. N Engl J Med. 2019; 381:1215-26.

6. Rosario M, French JL, Dirks NL, et al. Exposure-efficacy relationships for vedolizumab induction therapy in patients with ulcerative colitis or Crohn's disease. J Crohns Colitis. 2017; 11:921-9.

7. Yarur AJ, Jain A, Sussman DA, et al. The association of tissue anti-TNF drug levels with serological and endoscopic disease activity in inflammatory bowel disease: the ATLAS study. Gut. 2016; 65:249-55

8. Bowcutt R, Malter LB, Chen LA, et al. Isolation and cytokine analysis of lamina propria lymphocytes from mucosal biopsies of the human colon. J Immunol Methods. 2015; 421:27-35.

9. Paul S, Williet N, Di Bernado T, Berger AE, et al. Soluble Mucosal Addressin Cell Adhesion Molecule 1 and Retinoic Acid are Potential Tools for Therapeutic Drug Monitoring in Patients with Inflammatory Bowel Disease Treated with Vedolizumab: A Proof of Concept Study. J Crohns Colitis. 2018; 12:1089-1096.

10. Yoshihara T, Shinzaki S, Kawai S, et. al. Tissue Drug Concentrations of Anti-tumor Necrosis Factor Agents Are Associated with the Long-term Outcome of Patients with Crohn's Disease. Inflamm Bowel Dis. 2017; 23:2172-2179.

Appendix A Schedule of Study Procedures

**Week**	Screening	Week 0	Week 2	Week 6	Week 14	Week 22	Week 30	Week 38	Week 46	Week 54/Withdrwal[c]
**Day**[a]	-21~-1	1	15	43	99	155	211	267	323	379/Withdrwal
**Allowable range (Day)**		1	8~22	36~50	92~106	148~162	204~218	260~274	316~330	372~386/Withdrwal
**VISIT**	VISIT 1	VISIT 2	VISIT 3	VISIT 4	VISIT 5	VISIT 6	VISIT 7	VISIT 8	VISIT 9	VISIT 10/Withdrwal
Informed consent procedure [b]	×									
Demographics	×									
Medication history	×									
Physical examination		×	×	×	×	×	×	×	×	×
Weight, height and BMI	×									
Concomitant medications		×	×	×	×	×	×	×	×	×
Concurrent medical conditions	×									
Tissue and blood sampling for vedolizumab concentration					×					×
Tissue and blood sampling for α4β7 receptors occupancy measurement					×					×
Check of the patient diary			×	×	×	×	×	×	×	×
Complete Mayo score		×			×					×
Partial Mayo score			×	×		×	×	×	×	
Blood sampling for AVA		×			×					×
Tissue sampling for transcriptome analysis		×			×					×
Blood sampling for immuno-phenotyping		×			×					×
Biomarkers		×			×					×
Pretreatment events/AEs	×	×	×	×	×	×	×	×	×	×
Treatment compliance		×	×	×	×	×	×	×	×	

(a) The starting day of study treatment (day of the first dose) is designated as Day 1. The one day before the starting day is designated as Day -1

(b) Informed consent will be obtained, prior to all study procedures.

(c) The final visit will be performed at Week 54 (Visit 10) or at discontinuation.

Appendix B Special situation to be reported to Takeda

- Pregnancy: Any case in which a pregnancy patient is exposed to a Takeda Product or TAKEDA-IMP or in which a female patient or female partner of a male patient becomes pregnant following treatment with Takeda Product or TAKEDA-IMP. Exposure is considered either through maternal exposure or via semen following paternal exposure
- Breastfeeding: infant exposure from breast milk
- Overdose: All information of any accidental or intentional overdose
- Drug abuse, misuse or medication error: All information on medicinal product abuse, misuse or medication error (potential or actual)
- Suspected transmission of an infectious agent: All information on a suspected (in the sense of confirmed or potential) transmission of an infectious agent by a medicinal product.
- Lack of efficacy of Takeda Product and/or TAKEDA-IMP
- Occupational exposure: Cases of adverse reactions caused by exposure to ethical drugs in the profession. It does not include exposure to raw materials during the manufacturing process before release.
- Use outside the terms of the marketing authorization, also known as "off-label": Examples include intentional use of a product under conditions other than those described in the product label, such as a different indication/patient population (e.g. different age group)/route/dose.
- Use of falsified medicinal product
- Use of counterfeit medicinal product: Counterfeit or falsified medicine also includes any false representation of:

  ・Packaging and labelling; name or composition and strength of any ingredients including excipients

  ・Manufacturer, country of manufacture, country of origin, or supplier such as drug marketing authorization holder

  ・Records of distribution routes

  However, counterfeit drugs do not include unintended quality defects that do not infringe intellectual property rights.

Appendix C Responsibilities of the investigator

1. To appropriately conduct the study in compliance with this protocol and the Clinical Trials Act and with the highest respect for human rights, safety, and welfare of subjects.
2. To confirm the contents of this protocol and submit an agreement to the principal investigator and Takeda as evidence have agreed to this protocol. If the protocol is revised, submit an agreement in the same manner.
3. To prepare a list of any other investigators and/or study collaborators when certain important study-related activities are divided by investigators and/or study collaborators and submit the list to the principal investigator and/or Takeda as required.
4. To use the informed consent form approved by CRB.
5. To check the contents of the study contract.
6. To provide enough information on the protocol, drug and duties of each personnel to other investigators and study collaborators and give guidance and supervision.
7. To select subjects who satisfy the inclusion criteria, give explanation using written information, and obtain consent in writing.
8. To be responsible for all medical judgments related to the study.
9. When emergency reporting of SAEs, etc., is required, to immediately report it in writing to Takeda.
10. To maintain adequate and accurate source documents and trial records that include all pertinent observations on each of subjects. Source data should be attributable, legible, contemporaneous, original, accurate, and complete. Changes to source data should be traceable, and should not obscure the original entry.
11. To ensure that the (e)CRFs are accurate and complete, electronically sign, and submit them to the principal investigator or Takeda.
12. To verify any entries on the (e)CRFs made by the investigator or transcribed by the collaborator from source documents, electronically sign, and submit them to the principal investigator or Takeda.

Study Administrative Structure and Study Period

1. Study Administrative Structure

(1) Representative investigator

(2) Principle Investigator

(3) Supporting the research and development plan

(4) Practical coordination and management (research secretariat)

It is mainly responsible for operations related to study operation (Selection and contract of study sites, collection and transmission of safety information, handling of inquiries from study sites, progress management of this clinical research, etc.) in accordance with the separately prescribed “Operating Procedures.”

(5) Monitoring

It is mainly responsible for monitoring operations (Implementation of guidance for study sites, visit monitoring, etc.) in accordance with the separately prescribed “Operating Procedures.”

(6) Data Management

It is mainly responsible for data management operations (Establishment/operation of electronic case report form system, central monitoring, etc.) in accordance with the separately prescribed “Operating Procedures.”

(7) Statistical Analyses

It is mainly responsible for statistical analysis operations (Preparation of analysis datasets, preparation of analysis forms, etc.) in accordance with the separately specified “Operating Procedures.”

(8) Auditing organization

(9) Clinical Laboratory and Special Testing Institutions

1) Laboratory

2) Special laboratory

It is mainly in charge of inspection operations (Collection and measurement of samples, preparation of pathological samples, etc.) in accordance with the Outsourcing Contract with the person in charge of supporting the research and development plan.

2. Research period

Date of publication of jRCT to March 31, 2024
(From enrollment of the first subject to completion of the last subject: date of publication of the jRCT to March 31, 2023)

Medical institution and principal investigator

No.	Site	Job title	Principle Investigator	Address	TEL
1					
2					
3					

Conflict of Interest

- Article 21, item 1 of the Regulations: Provision of research funds, etc. and other involvement by pharmaceutical manufacturers and distributors: Yes
- Article 21, item 2 of the Regulations: Provision of donations, compensation for manuscript writing, lectures, and other work by Takeda to the principal investigator, co-investigator, statistical analysis manager, and other persons listed in the research plan who will clearly benefit from the implementation of the clinical research (hereinafter referred to as "conflict of interest declarers"), and other involvement (limited to involvement in the year of the declaration of the conflict of interest and the previous year): No
